# Fatty acid export (FAX) proteins contribute to oil production in the green microalga *Chlamydomonas reinhardtii*

**DOI:** 10.3389/fmolb.2022.939834

**Published:** 2022-08-30

**Authors:** Janick Peter, Marie Huleux, Benjamin Spaniol, Frederik Sommer, Jens Neunzig, Michael Schroda, Yonghua Li-Beisson, Katrin Philippar

**Affiliations:** ^1^ Plant Biology, Center for Human- and Molecular Biology (ZHMB), Saarland University, Saarbrücken, Germany; ^2^ Aix Marseille Univ, CEA, CNRS, Institute of Bioscience and Biotechnology of Aix Marseille, BIAM, Saint Paul-Lez-Durance, France; ^3^ Molecular Biotechnology and Systems Biology, TU Kaiserslautern, Kaiserslautern, Germany

**Keywords:** chloroplast, endoplasmic reticulum, fatty acid transport, microalgae, oil production

## Abstract

In algae and land plants, transport of fatty acids (FAs) from their site of synthesis in the plastid stroma to the endoplasmic reticulum (ER) for assembly into acyl lipids is crucial for cellular lipid homeostasis, including the biosynthesis of triacylglycerol (TAG) for energy storage. In the unicellular green alga *Chlamydomonas reinhardtii*, understanding and engineering of these processes is of particular interest for microalga-based biofuel and biomaterial production. Whereas in the model plant *Arabidopsis thaliana*, FAX (fatty acid export) proteins have been associated with a function in plastid FA-export and hence TAG synthesis in the ER, the knowledge on the function and subcellular localization of this protein family in Chlamydomonas is still scarce. Among the four FAX proteins encoded in the Chlamydomonas genome, we found Cr-FAX1 and Cr-FAX5 to be involved in TAG production by functioning in chloroplast and ER membranes, respectively. By *in situ* immunolocalization, we show that Cr-FAX1 inserts into the chloroplast envelope, while Cr-FAX5 is located in ER membranes. Severe reduction of Cr-FAX1 or Cr-FAX5 proteins by an artificial microRNA approach results in a strong decrease of the TAG content in the mutant strains. Further, overexpression of chloroplast Cr-FAX1, but not of ER-intrinsic Cr-FAX5, doubled the content of TAG in Chlamydomonas cells. We therefore propose that Cr-FAX1 in chloroplast envelopes and Cr-FAX5 in ER membranes represent a basic set of FAX proteins to ensure shuttling of FAs from chloroplasts to the ER and are crucial for oil production in Chlamydomonas.

## Introduction

In all living organisms, fatty acids (FAs) are essential building blocks for polar, membrane-building structural lipids and energy-storage acyl lipids, mainly represented by neutral triacylglycerol (TAG) molecules that accumulate in lipid droplets. In photosynthetically active eukaryotes, ranging from algae to land plants, *de novo* synthesis of FAs takes place in the plastid stroma (Troncoso-Ponce et al., 2015; Li-Beisson et al., 2019). After synthesis, FAs are assembled into acyl lipids either in plastids *via* the so-called prokaryotic pathway or in the endoplasmic reticulum (ER) by the eukaryotic pathway. In the ER, all phospholipids for non-plastid, cellular lipid bilayer membranes, as well as precursors for complex extracellular lipophilic compounds in land plants are produced [for an overview on algal/plant lipid metabolism *see* (Li-Beisson et al. (2013), Li-Beisson et al. (2015), Lavell and Benning (2019))]. Further, ER membranes are the site for the biogenesis of lipid droplets (LDs). These are filled with TAG storage oils that have been assembled from glycerol and three acyl chains, which have been delivered and shuttled from the plastid (Bates, 2016; Xu and Shanklin, 2016; Li-Beisson et al., 2019; Li-Beisson et al., 2021). Thus, for a proper function of cellular lipid homeostasis in plant and algal cells, transport and distribution of lipophilic compounds are indispensable and mediated by membrane transport proteins (Li et al., 2016; Li-Beisson et al., 2017; Lavell and Benning, 2019) as well as *via* membrane contacts between organelles or vesicular traffic (Hurlock et al., 2014; Block and Jouhet, 2015; Michaud and Jouhet, 2019).

The green, unicellular alga *Chlamydomonas reinhardtii* in the past two decades has emerged as model organism for studying photosynthesis, flagella, carbon metabolism and more recently for dissecting molecular mechanisms of TAG synthesis and storage in LDs (Merchant et al., 2012; Scranton et al., 2015; Takeuchi and Benning, 2019). Thus, in the recent years, a focus was on understanding and engineering FA and TAG biosynthesis as well as LD biogenesis and disassembly in Chlamydomonas cells (Blatti et al., 2013; Kim et al., 2018; Gu et al., 2021; Li-Beisson et al., 2021). Since plant lipid transport is best understood in *Arabidopsis thaliana* (Li et al., 2016; Li-Beisson et al., 2017; LaBrant et al., 2018; Lavell and Benning, 2019), this model plant serves as blueprint for studies in Chlamydomonas, especially for subcellular distribution and transport of FAs from plastids to the ER. In the ER membrane, primary active ABC transporters that mediate uptake of FAs and/or acyl-CoA into the ER lumen in Arabidopsis (Kim et al., 2013) and in Chlamydomonas (Jang et al., 2020) have been described. Both proteins, which belong to the same ABC transporter subfamily, are crucial for the accumulation of TAGs in seed tissue (At-ABCA9) and Chlamydomonas cells (Cr-ABCA2). For the export of FAs from plastids, members of the FAX protein family have been pinpointed since the discovery of FAX1 in Arabidopsis (Li et al., 2015). At-FAX1 inserts with four membrane-intrinsic α-helices into the inner envelope (IE) membrane of chloroplasts and is able to mediate FA transport across lipid bilayer membranes. Further, our detailed study of At-FAX1 knockout and overexpressing lines in Arabidopsis revealed that the function of FAX1 is important for cellular lipid homeostasis, e.g. for ER-produced TAG oils and phospholipids, cuticular wax composition and lipophilic biopolymers of the outer pollen cell wall (Li et al., 2015). In addition, it was shown that seed-specific overexpression of At-FAX1 increases seed oil content in Arabidopsis and that the FAX1 ortholog in *Brassica napus* contributes to seed oil production, as well (Tian et al., 2018; Xiao et al., 2021). Thus, in land plants, the function of plastid IE-intrinsic members from the FAX-protein family is clearly associated with a role in export of FAs from plastids and hence affects the homeostasis of lipid compounds throughout plant development (Li et al., 2015; Tian et al., 2018; Tian et al., 2019; Li et al., 2020; Zhu et al., 2020; Cai et al., 2021; Huang et al., 2021; Xiao et al., 2021).

In unicellular algae, potential plastid envelope FAX-proteins have been analyzed in the red and green algal model systems *Cyanidioschyzon merolae* (Takemura et al., 2019) and *Chlamydomonas reinhardtii* (Li et al., 2019), respectively. The protein Cm-FAX1 in *C. merolae* most likely represents the ortholog to Arabidopsis At-FAX1 and was localized to plastid envelopes by indirect immuno-fluorescence microscopy on *C. merolae* cells overexpressing a FLAG-tagged Cm-FAX1 protein (Takemura et al., 2019). Further, Takemura and co-workers (2019) could show that a Cm-FAX1 null mutant in comparison to wild-type cells has higher free fatty acid (FFA) content. In a Cm-FAX1 overexpressor strain, however, the FFA level was reduced but the amount of TAG storage lipids increased by about 2.4-fold. In Chlamydomonas, two FAX-like proteins, named Cr-FAX1 and Cr-FAX2 were examined by Li et al. (2019). Although any data on subcellular localization of these proteins is missing, the authors conclude that both have similar functions to At-FAX1 and are involved in export of FAs from chloroplasts to the cytosol in Chlamydomonas cells. This hypothesis is purely based on some conserved sequence motifs between At-FAX1 and the two Cr-FAX proteins, and the finding that alleged overexpressing strains of Cr-FAX1 and Cr-FAX2 accumulate more TAG than wild type and affect FA as well as polar lipid homeostasis (Li et al., 2019). The data presented by Li et al. (2019) is somewhat disappointing since the overexpression of both FAX proteins is only followed in one single strain per protein and was analyzed only at the transcript level. The increase in transcript content in overexpression strains compared to wild type was small, i.e. around 1.5- and 1.3-fold for Cr-FAX1 and Cr-FAX2, respectively (Li et al., 2019). Although proteins of the Arabidopsis FAX5/6 and FAX7 subfamilies are predicted to be in ER and/or secretory pathway membranes (Li et al., 2015), so far no function of FAX5/6 and FAX7 proteins associated with FA/lipid transport and homeostasis has been described in the green lineage. As part of our long-term effort in understanding FA and lipid transport in green photosynthetic cells, here we have investigated in detail the subcellular localization and impact on lipid homeostasis of two distinct groups of FAX proteins - i.e. FAX1 and FAX5/6 - in the green microalga Chlamydomonas.

## Materials and methods

### Strains and culture conditions

*Chlamydomonas reinhardtii* strain cw15-325 (cw_d_ *mt* ^*+*^ *arg7 nit1* ^*+*^ *nit2* ^*+*^) was used for transformation with amiRNA constructs (containing the *ARG7* gene for selection). UVM4, which is derived from cw15-302 (cw_d_ *mt* ^*+*^ *arg7 nit1* ^*-*^ *nit2* ^*-*^), was used for immunolocalization as well as overexpression of Cr-FAX1 and Cr-FAX5 due to reduced transgene suppression (Neupert et al., 2009). Cultures were grown mixotrophically in Tris–acetate-phosphate (TAP) medium (Kropat et al., 2011) on a rotary shaker (140 rpm) at 20°C and continuous light (30 µmol photons m^−2^ s^−1^). Growth was followed by determining the optical density of cultures at 750 nm (OD_750_) and the cell number (Supplementary Figure S1). For growing cw15-325 cells prior to transformation, arginine (100 μg/ml) was added to the medium. For lipid analyses, cells were cultivated at 25°C with constant continuous light (80–100 µmol photons m^−2^ s^−1^) in TAP liquid medium in conical glass flasks in incubators (Multitron, Infors HT) shaking at 120 rpm. Exponentially grown cells were counted with a Multisizer 4 (Beckman Coulter), and a fixed number of cells was harvested by centrifugation. Chlamydomonas strains were kept on TAP agar plates under constant light at 20–25°C in a culture room.

### Generation of artificial microRNA constructs

**FIGURE 1:**
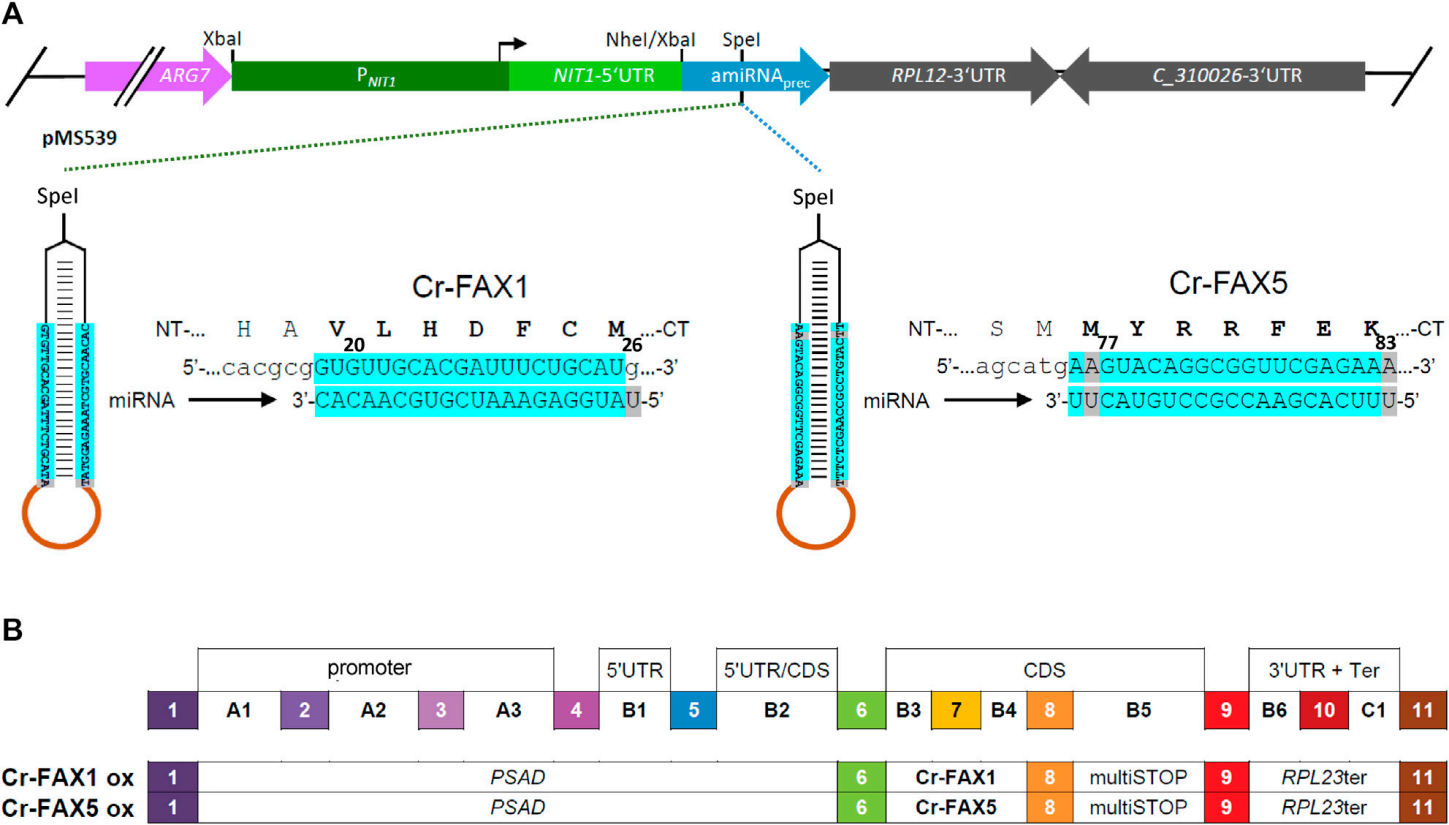
Knockdown and overexpression constructs for *Cr-FAX1* and *Cr-FAX5* genes. **(A)** Schematic drawing of artificial microRNA (amiRNA) constructs for knockdown of Cr-FAX1 and Cr-FAX5. The amiRNAs directed against Chlamydomonas *Cr-FAX1* (left) and *Cr-FAX5* genes (right) are *SpeI*-subcloned into the pMS539 plasmid vector (top), which harbors the inducible *NIT1* (nitrate reductase 1) promoter (for details see Schmollinger et al., 2010). The amiRNA generated by pMS539 targets regions in the second exons of *Cr-FAX1* (left) and *Cr-FAX5* (right), coding for amino acids 20–26 of mature Cr-FAX1 and 77–83 of Cr-FAX5, respectively. Base-pairing nucleotides of the amiRNA and *Cr-FAX1*, *Cr-FAX5* mRNAs are shaded in turquoise. **(B)** Schematic drawing of level 1 constructs according to the MoClo syntax (Patron et al., 2015; Crozet et al., 2018) for overexpression of *Cr-FAX1* and *Cr-FAX5* genes. The following level 0 modules were assembled in destination vector pICH47742 (Weber et al., 2011). PSAD, promoter of photosystem I reaction center subunit II (position A1-B2); respective *FAX* gene (position B3-B4); multiSTOP sequence (B5); *RPL23*ter: terminator of 50S ribosomal protein L23 (B6-C1).

The artificial microRNAs (amiRNAs) targeting Chlamydomonas *Cr-FAX1* and *Cr-FAX5* transcripts were designed with the WMD3 Web tool (Ossowski et al., 2008). The resulting oligonucleotides CrFAX1-amiFor, CrFAX1-amiRev, CrFAX5-amiFor, CrFAX5-amiRev (Supplementary Table S1) directed against the second exon of the respective *FAX* sequence (*see* Figure 1A), were annealed by boiling and slowly cooling-down in a thermocycler. The resulting DNA constructs were *Spe*I-subcloned into the pMS539 plasmid vector, which harbors the inducible *NIT1* (nitrate reductase 1) promoter (Schmollinger et al., 2010). Screening for correct constructs was done as described by (Molnar et al., 2009) and verified by sequencing. One microgram plasmid DNA was transformed into strain cw15-325 using the glass beads method (Kindle, 1990).

To evaluate the inducibility of the *NIT1* promoter for knockdown of Cr-FAX1 and Cr-FAX5, the respective Chlamydomonas strains were grown to mid-log phase (OD_750_ between 0.3 and 0.5) in TAP medium containing 7.5 mM NH_4_Cl as nitrogen source. For induction of the *NIT1* promoter, the nitrogen source was switched from ammonium to nitrate. To this end, cells were pelleted by centrifugation for 3 min at 1,500 *g* and 4°C. The supernatant was discarded, and the cells were washed twice with TAP medium containing 7.5 mM KNO_3_ as nitrogen source. Subsequently, growth continued in TAP-nitrate medium for up to 6 days.

### Generation of FAX overexpression and mVenus constructs by modular cloning (MoClo)

Genomic DNA from Chlamydomonas was isolated as described in Spaniol et al. (2022). The genomic sequences for *Cr-FAX1* and *Cr-FAX5* were amplified by PCR and “domesticated” by removing endogenous, internal *Bbs*I and *Bsa*I restriction sites and introducing *Bbs*I restriction sites at the 5′ and 3′ ends by PCR-based mutagenesis (for oligonucleotides *see* Supplementary Table S1). The respective PCR products were cloned into the recipient plasmid pAGM1287 by adding *Bbs*I and T4 DNA ligase (Weber et al., 2011), resulting in level 0 constructs for *Cr-FAX1, Cr-FAX5*. According to the MoClo syntax (Patron et al., 2015; Crozet et al., 2018), the FAX genes were inserted at positions B3-B4. For level 1 constructs, the respective *FAX* gene, the *PSAD* promoter (position A1-B2), multiSTOP sequence (B5), *RPL23* terminator (B6-C1), and the destination vector pICH47742 (Weber et al., 2011) were directionally assembled with *Bsa*I and T4 DNA ligase (*see* Figure 1B). For mVenus fluorescent constructs in the chloroplast stroma and ER lumen, we assembled the following level 1 modules in pICH47742 (*see* Supplementary Figure S4B). Chloroplast targeted mVenus: *HSP70A-RBCS2* hybrid promoter (A1-B1); the chloroplast transit peptide of universal stress protein A (USPA; B2); mVenus(i2), harboring the second intron of RBCS, plus stop codon (B3-B5); and the *RPL23* terminator (B6-C1). ER-targeted mVenus: *HSP70A-RBCS2* hybrid promoter (A1-B1); the signal peptide of BiP2 (B2); mVenus(i2), harboring the second intron of RBCS (B3-B4); the 3XHA+KDEL (ER retention signal) sequence (B5); and the *RPL23* terminator (B6-C1).

Subsequently, the respective level 1 module was combined with a level 1 construct harboring the *aadA* gene conferring resistance to spectinomycin, a proper end-linker, and the destination vector pAGM4673 (Weber et al., 2011), digested with *BbsI* and ligated by T4 DNA ligase in order to obtain the final level 2 device for transformation. Transformations were carried out with the *Chlamydomonas reinhardtii* strain UVM4 with 1 µg plasmid DNA of the respective level 2 device using the glass beads method (Kindle, 1990). Selection of transformants was performed on TAP agar medium containing 100 μg/ml spectinomycin.

### Isolation of proteins, membranes, and organelles

For the extraction of total cellular proteins, Chlamydomonas cells from 5–10 ml cultures were collected in mid-log growth phase (OD_750_ between 0.3 and 0.5, *see* Supplementary Figure S1) by centrifugation for 3 min at 1,500 g and 4°C. The cell pellet was resuspended in 60 µl DTT-carbonate buffer (0.1 M DTT, 0.1 M Na_2_CO_3_), to generate a homogenous solution prior to freezing at −20°C. After thawing, 55 µl SDS-sucrose buffer (5% [w/v] SDS, 30% [w/v] sucrose) was added and the sample was mixed thoroughly. Samples were then incubated at 99°C for 2 min, followed by 2 min on ice and insoluble material was removed by centrifugation at 16,000 *g* for 2 min. Next, the chlorophyll concentration of the supernatant, containing total cellular proteins, was determined. Chlorophyll was extracted with 80% acetone for 5 min on ice. After centrifugation at 16,000 *g* for 5 min, the absorption was measured at 645 and 663 nm. The chlorophyll concentration was calculated according to Porra and Scheer (1989): Chlorophyll [µg/µl] = [(A645 × 17.76) + (A663 × 7.34)].

For the isolation of total cellular membranes, cells were collected from 25 ml cultures grown to mid-log growth phase (3–5*10^6^ cells/ml) by centrifugation for 5 min at 3,100 *g* (swing out buckets) and 4°C. The supernatant was discarded, and the cell pellet was resuspended in 1 ml lysis buffer (10 mM Tris-HCl, pH 8.0, 1 mM EDTA, 0.25XcOmplete protease inhibitor cocktail [Roche]). 200 µl were directly used as the whole cell protein fraction for immunoblot analysis. The remaining cells were broken up by four cycles of freezing and thawing (liquid nitrogen and room temperature, respectively) and cellular membranes were pelleted by centrifugation for 30 min at 21,000 *g* and 4°C. The pellet, containing cellular membrane-intrinsic proteins was resuspended in 800 µl lysis buffer. The corresponding supernatant contained soluble cellular proteins and membrane vesicles of light density such as microsomes. The protein content of all fractions was determined by Bradford and BCA assays.

Chloroplast and microsomal membranes were isolated according to Jang et al. (2020). Therefore, cells were collected from 100 ml in mid-log growth phase (OD_750_ = 0.4–0.55, *see* Supplementary Figure S1) by centrifugation for 3 min at 1,500 *g* and 4°C. The cell pellet was resuspended in 40 ml homogenization buffer (250 mM sorbitol, 50 mM Tris-acetate pH 7.5, 1 mM EGTA-Tris pH 7.5, 2 mM DTT, 1XcOmplete protease inhibitor cocktail [Roche]). The suspension was kept at 4°C during homogenization by pulse sonication (UP50H [Hielscher, Germany]: cycle 0.4, intensity 20%). Subsequently, 20 ml of cell homogenate were subjected to serial centrifugation steps at 4°C, which precipitated non-broken cells and nuclei (500 *g*, 10 min), chloroplasts (3,000 *g*, 10 min), smaller organelles like mitochondria/peroxisomes (20,000 *g*, 30 min) and microsomes (100,000 *g*, 4 h, swing out rotor). The respective chloroplast and microsomal membrane pellets were each resuspended in 500 µl homogenization buffer and the protein content was determined by a BCA assay.

### Immunoblot analysis

**FIGURE 2:**
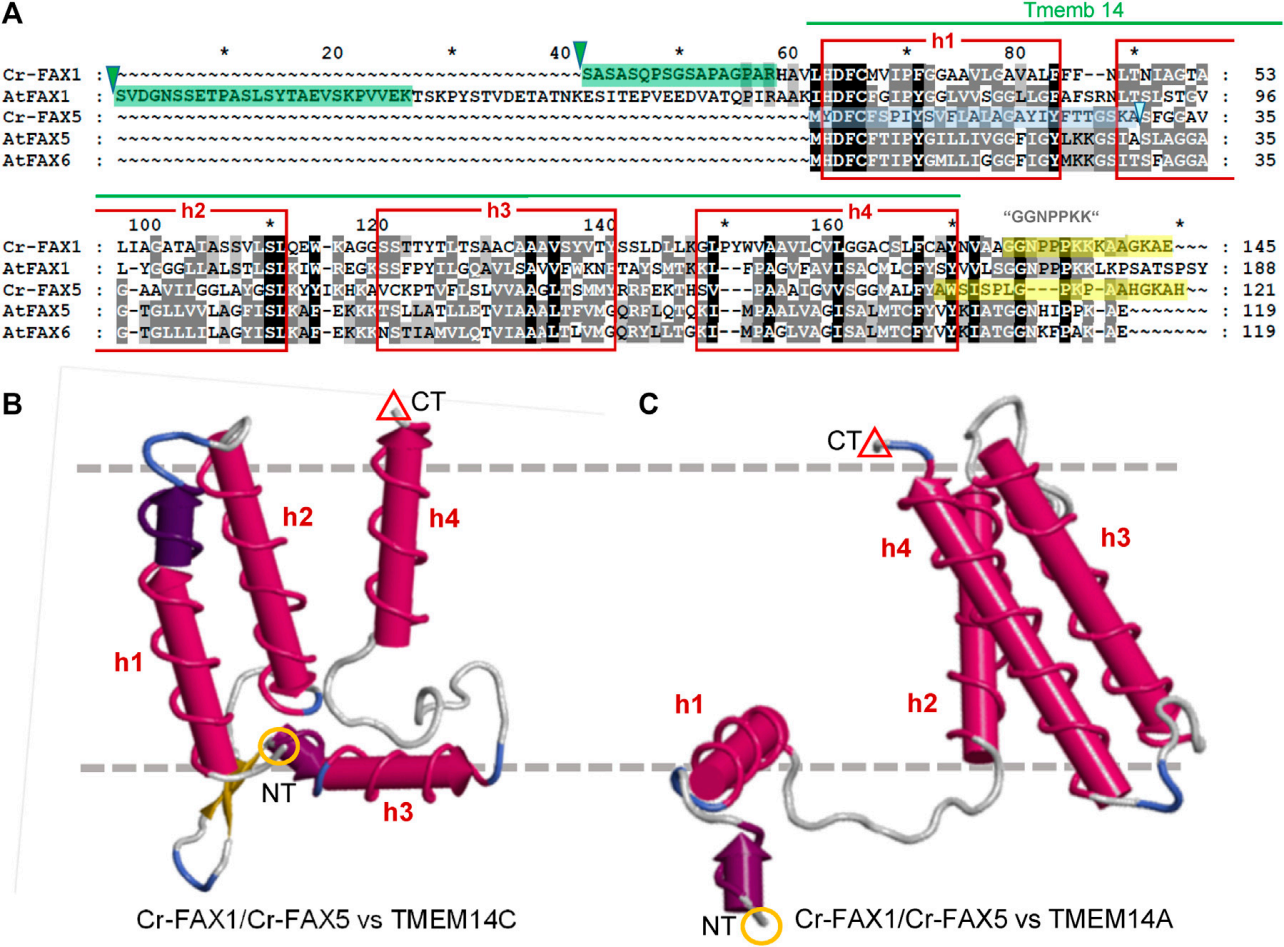
FAX1 and FAX5/6 proteins in Chlamydomonas and Arabidopsis. **(A)** Sequence alignment of mature FAX1 and FAX5/6 proteins in Chlamydomonas and Arabidopsis. The four membrane-embedded α-helices (red boxes) within the Tmemb_14 domain (pfam PF03647 motif, green line) are depicted according to the Aramemnon consensus prediction AramTmCon for At-FAX1 (Schwacke et al., 2003). Oligopeptides used to generate antisera against Cr-FAX1 and Cr-FAX5 are specified by yellow boxes. Processing sites for stromal peptidases according to TargetP2.0 (Almagro Armenteros et al., 2019) are indicated by green triangles, the presumed ER signal peptide for Cr-FAX5 is depicted by a blue box. The most N-terminal peptides of mature Cr-FAX1 and At-FAX1, identified after peptide sequencing are highlighted by green boxes. Among the FAX-protein family in Arabidopsis, Cr-FAX1 shows highest similarity to At-FAX1 (26% aa identity) and Cr-FAX5 is most likely related to At-FAX5 (32% identical aas). Names, gene codes (AGI, Phytozome), and protein IDs (UniProt) are as follows: Cr-FAX1 (Cre10.g421750, A8ICM7), At-FAX1 (At3g57280, Q93V66), Cr-FAX5 (Cre08.g366000, A8J403), At-FAX5 (At1g50740, Q9C6T7), At-FAX6 (At3g20510, Q9LJU6). Note that our Cr-FAX1 and Cr-FAX5 correspond to CrFAX1 and CrFAX2, described in (Li et al., 2019), respectively. **(B, C)** Structural modelling of Cr-FAX1 and Cr-FAX5 (Phyre2; Kelley et al., 2015) revealed high similarity to the structure of human TMEM14C and TMEM14A proteins (confidence 99.7–99.9%). Depicted are 3D models of **(B)** Cr-FAX5 versus TMEM14C (PDB entry c2losA) and **(C)** versus TMEM14A (PDB entry c2lopA; right) generated by *FirstGlance* in *Jmol* (firstglance.jmol.org). Note that models of Cr-FAX1 (not shown) are highly similar to those of Cr-FAX5. The four α-helices (h1–h4) of the Tmemb_14 domain of FAX/TMEM14 proteins are depicted as red cylinders. N- and C-terminal ends of the proteins are highlighted by orange circles (NT) and red triangles (CT). Boundaries of a lipid bilayer membrane are indicated by gray, dashed lines.

For immunoblot analyses, proteins from Chlamydomonas cells, organelles and membranes were separated by SDS-PAGE and transferred to PVDF membranes. Primary antisera were used in 1:1,000 dilution in TTBS buffer (100 mM TRIS-HCl, pH 7.5, 150 mM NaCl, 0.2% Tween-20, 0.1% BSA). Secondary anti-rabbit IgG horseradish peroxidase (Santa Cruz Biotechnology) was diluted 1:10,000 in TTBS. Blots were stained in ECL solution (Pierce ECL Western Blotting Substrate, Thermo Scientific) according to the manufacturer’s instructions and chemiluminescent signals were detected by the iBright1500 imaging system (Invitrogen). Cr-FAX1 and Cr-FAX5 antisera were raised in rabbit (Pineda Antibody Service, Berlin, Germany) against C-terminal peptide sequences of both proteins (*see* Figure 2A). For controls of chloroplast and microsomal fractions we used the following antisera, generated in rabbit: The antiserum against BiP luminal-binding protein (rabbit antibody, product No. AS09 481) was purchased from Agrisera (Sweden), α-Cr-ABCA2, directed against the ER-localized ABC transporter ABCA2 (Jang et al., 2020) was provided by Prof. Y. Lee, α-Lhcb4 for detection of thylakoid CP29 chlorophyll a/b binding protein of PSII from higher plants has been described previously (Duy et al., 2007).

For the detection of N-terminal peptides in mature Cr-FAX1 and At-FAX1 proteins, peptide sequencing of SDS gel slices of the same size as of the respective bands stained by antisera was carried out. To this end, a tryptic digest of PAGE separated proteins and mass spectrometric analysis was performed as described in Rütgers et al. (2017) on a TT6600 Instrument (AB Sciex). MS data analysis was performed with MaxQuant software v 1.6.0.1 using default settings including phospho-STY variable modifications and searching against proteins from *Arabidopsis thaliana* and *Chlamydomonas reinhardtii* derived from the UniProt database.

### Immunofluorescence assays

UVM4 cells from 1 ml TAP medium culture grown to the mid-log phase (∼5*10^6^ cells/ml, *see* Supplementary Figure S1) were fixed on poly-L-lysin coated Poly-Prep slides (Sigma). Positioning of chloroplasts and nuclei was determined visually by screening through images with a confocal laser scanning microscope (Leica LSM780). Subsequently, slides were dipped for 2 min in cold methanol (−20°C), followed by rinsing five times with phosphate-buffered saline (PBS: 27 mM NaCl, 2.7 mM KCl, 10 mM Na_2_HPO_4_, 1.8 mM KH_2_PO_4_, pH 7.4). Blocking of unspecific signals was achieved by incubation with 1% BSA in PBS for 30 min, again followed by rinsing five times with PBS solution. Primary antisera against Cr-FAX1 (1:1,000), Cr-FAX5 (1:1,000) or hemagglutinin (HA; 1:500; Agrisera, Sweden) were applied in PBS + 1% BSA for 3 h, followed by rinsing five times with PBS solution. Subsequently, all steps were performed in darkness and fixed cells were incubated with the secondary antibody anti-rabbit IgG-TRITC (IgG-tetramethylrhodamine-isothiocyanate; Sigma), diluted 1:250 in PBS + 1% BSA for 1 h. Fixed cells were washed five times with PBS solution before being overlayed with Prolong Gold antifade reagent (Invitrogen). Pseudo transmission images (PTI) as well as TRITC fluorescence signals (excitation at 561 nm, emission from 565 to 583 nm) were collected with an LSM780.

As controls for chloroplast stroma and ER lumen, signals from Chlamydomonas cells expressing mVenus fluorescent constructs were recorded. For this, the respective strains were grown to mid-log phase in TAP medium and immobilized by adding 2.5% glutaraldehyde. By centrifugation at 1,500 *g*, cells were concentrated and placed on a glass slide. Signals for mVenus fluorescence were collected from 520 to 564 nm with a confocal laser scanning microscope (Leica LSM780) after excitation at 512 nm. The laser line at 458 nm was used for excitation of chlorophyll signals, which were collected from 626 to 735 nm.

### Lipid extraction and analyses

Total lipids were extracted from Chlamydomonas cells using a protocol based on isopropanol and methyl-tert-butyl ether (MTBE) extraction as detailed in Legeret et al. (2016). Briefly, cells were harvested in glass tubes by centrifugation at 3,200 *g*, 5 min at 4°C. Hot isopropanol (1 ml) containing 0.01% (w/v) butylated hydroxytoluene (BHT; pre-heated to 85°C) were added to the cell pellet, vortexed and heated up for 10 min at 85°C. Once cooled down, MTBE and water were added to reach a final ratio of isopropanol/MTBE/water of 1:3:1 (v/v/v). The mixture was vortexed and then phase separated by centrifugation at 4°C for 2 min at 3,200 *g*. The organic upper phase was transferred to a clean glass tube using a Pasteur pipette. An additional volume of MTBE was added to the cell pellet to extract remaining lipids, and again the upper phase collected after a brief centrifugation. The combined organic phases were then evaporated under a gentle stream of N_2_ and re-dissolved in a mixture of chloroform/methanol (2:1, v/v) and kept at −20°C until further analysis.

TAGs and polar lipids were quantified using a densitometry method after being separated from each other using Thin Layer Chromatography (TLC) techniques. An ATS5 autosampler (Camag Switzerland) was used to deposit lipid extracts to a silica TLC plate made of silica gel 60 F254 (Merck KGaA, Germany). Lipid classes were then separated by developing the plate in an ADC2 automatic developing chamber (Camag) using a hexane/diethyl ether/acetic acid (17/3/0.2, v/v/v) solvent mixture for TAG analysis or acetone/toluene/water (91/30/8, v/v/v) solvent mixture for polar lipid analysis. TLC plates were thoroughly dried under the hood, before being dipped for 6 s in a solution containing CuSO_4_ reagent (20 g CuSO_4_, 200 ml methanol, 8 ml H_2_SO_4_, 8 ml H_3_PO_4_), heated at 141°C for 30 min on a TLC plate heater and finally scanned using a TLC Scanner 3 with WinCATs software (Camag). TAG or polar lipids were then quantified by comparing to a curve generated with corresponding lipid standard (Sigma-Aldrich, Saint-Louis, United States; Larodan Fine Chemicals AB, Malmö, Sweden). Lipid standards used were triheptadecanoin (C17:0 TAG, Sigma-Aldrich, Saint-Louis, United States), monogalactosyl-distearoylglyceride (MGDG; Larodan Fine Chemicals AB, Malmö, Sweden), digalactosyl-distearoylglyceride (DGDG; Larodan Fine Chemicals AB), 1,2-dipalmitoyl-*sn*-glycerol-3-phospho-(1′-rac-glycerol) (PG; Avanti Polar Lipids, AL, United States) and 1,2-dipalmitoyl-*sn*-glycerol-3-phospho-ethanolamine (PE; Avanti Polar Lipids).

For fatty acid composition analysis, a fraction of the extracted lipids or whole cells were converted to fatty acid methyl esters (FAMEs) using an acid-based transmethylation. Briefly, to cell pellet or total lipid extracts, 1 ml of 5% H_2_SO_4_ in methanol was added in a glass tube with a Teflon-lined screw cap. In addition, we added 0.01% BHT (final concentration) and 250 µl of toluene to improve solubility when TAG content is high. The internal standard used was triheptadecanoin (C17:0 TAG). The mixture was heated up at 85°C for 1.5 h. Once cooled down, hexane and 1 ml of 0.9% NaCl solution was added to extract FAMEs and allow phase separation. After centrifugation, the upper organic phase was transferred to a new tube and dried under a stream of N_2_, then analyzed by a GC-MS detailed in Legeret et al. (2016).

All lipid data were analyzed by student t-test (double sided *p*-value: ***p < 0.001; **p < 0.01; *p < 0.05).

## Results

### Chlamydomonas contains four FAX proteins with distinct structural features

When we examined publicly available databases, we identified four FAX-like proteins in *Chlamydomonas reinhardtii* (Table 1). According to TargetP 2.0 predictions (Almagro Armenteros et al., 2019), the proteins encoded by Cre10.g421750 and Cre08.g383300 are in chloroplasts and possess cleavable, N-terminal transit peptides of 54 and 37 amino acids (aa), respectively. In contrast, the protein corresponding to Cre08.g366000 is expected to hold an N-terminal signal peptide of 29 aa for the ER, and no prediction for subcellular targeting of the gene product from Cre09.g387838 was possible (Table 1). All four Cr-FAX proteins are annotated to belong to the FAX/TMEM14 protein family (PANTHER “transmembrane protein 14” subfamily, PTHR12668) and contain the FAX-like Tmemb_14 domain (Pfam entry PF03647) with four conserved α-helical domains (Figure 2 and Supplementary Figure S2A). In comparison with sequences and motifs of the plant FAX protein family, we found that the chloroplast predicted Cre10.g421750 and Cre08.g383300 are most similar to FAX1- and FAX3-subfamilies, respectively (Supplementary Figure S2). Thus, in the following we refer to Cre10.g421750 as Cr-FAX1 and to Cre08.g383300 as Cr-FAX3 (Table 1). The protein encoded by Cre08.g366000 we named Cr-FAX5, since it most likely represents the ortholog to At-FAX5, and Cre09.g387838/Cr-FAX7 corresponds to At-FAX7 (Figure 2 and Supplementary Figure S2). Note that Cr-FAX1 is identical to the protein named CrFAX1 by Li et al. (2019). For our Cr-FAX5, however, Li and coworkers chose the name CrFAX2, although no similarity to the seed-plant and plastid-specific FAX2 proteins [*see* Figure 2A; Supplementary Figure S2B and Tian et al. (2019), Li et al. (2020)] can be found.

**TABLE 1:** FAX proteins in *Chlamydomonas reinhardtii*.

name	Gene	Protein-ID	Length [aa]	MW [kDa]	Pre-loc	At ortholog [id/sim aa]
**Cr-FAX1** *	Cre10.g421750	A8ICM7	199 (**145**)	19.9 (**14.3**)	**C**	At-FAX1 [26/37%]
Cr-FAX3	Cre08.g383300	A0A2K3DI83	208 (171)	19.7 (15.7)	C	At-FAX3 [24/38%]
**Cr-FAX5** *	Cre08.g366000	A8J403	**121** (92)	**12.7** (9.5)	**ER**/SP	At-FAX5 [32/51%]
Cr-FAX7	Cre09.g387838	A0A2K3DDV7	109	11.2	-	At-FAX7 [28/47%]

The Chlamydomonas genome harbors four genes encoding for FAX-like proteins. Listed are names, genes (Phytozome; Goodstein et al., 2012), and protein-IDs (UniProtKB), length in amino acids (aa), molecular weight in kDa and the predicted subcellular localization (TargetP 2.0; Almagro Armenteros et al., 2019). In brackets are values for the respective predicted mature proteins; bold letters indicate proteins and verified features of this study. The similarity to the presumed Arabidopsis ortholog (*see* also Supplementary Figure S2B) is given in % identical (id) and similar (sim) aa. *: Note that Cr-FAX1/Cre10.g421750 and Cr-FAX5/Cre08.g366000 correspond to CrFAX1 and CrFAX2, described in Li et al. (2019), respectively. C, chloroplast; ER, endoplasmic reticulum; SP; secretory pathway; -, no location can be predicted.

The mature Cr-FAX1 protein is predicted to have 14.3 kDa and within the Tmemb_14 domain contains the typical peptide stretches identified for the plant FAX1 subfamily (Figure 2A). Besides the confirmed cleavage site for a chloroplast stromal peptidase, in particular the C-terminal “GGNPPKK” motif and the highly conserved sequence in the first α-helix classify Cr-FAX1 to be a FAX1-like protein. Structural modelling revealed that like for At-FAX1 [*see* Li et al. (2015)], the structure of Cr-FAX1 with high similarity fits to that documented for the human TMEM14C and TMEM14A proteins (Figures 2B,C; Klammt et al., 2012). Thus, most likely the third α-helix of Cr-FAX1 has an amphiphilic character and orients perpendicular to the lipid bilayer membrane. Analogous to At-FAX1, also the first helix of Cr-FAX1 shows amphiphilic character due to conserved hydrophilic residues at the N-terminal end and thus might be somewhat tilted vertically inside the membrane. Further, this first α-helix of the Tmemb_14 domain of Cr-FAX1 is not only highly similar to At-FAX1, but also to Cr-FAX5 and At-FAX5/At-FAX6, another conserved feature of plant FAX1- and also of FAX5/6-subfamilies. The FAX5/6 subfamily is conserved throughout plant species, but not always represented by two gene copies like for the presumed paralogs At-FAX5, At-FAX6 in Arabidopsis (Könnel et al., 2019). Cr-FAX5 is most similar to At-FAX5 (Table 1) and in the green microalga Chlamydomonas represents the only FAX5/6-like protein that could be identified. In consequence, we classify Cr-FAX5 to be the Chlamydomonas member of the plant FAX5/6 group. The molecular mass of Cr-FAX5 is calculated to be 12.7 kDa and an N-terminal ER signal-peptide that includes the first α-helix of the Tmemb_14 domain is predicted (Table 1 and Figure 2). Again, the topology of Cr-FAX5 can be modeled to that of hm-TMEM14C and TMEM14A (Figures 2B,C). We therefore conclude that Cr-FAX1 and Cr-FAX5 have a very similar topology inside the lipid bilayer membrane: *1*) When modeled according to hm-TMEM14C the third α-helix of Cr-FAX1 and Cr-FAX5 represents a “classical,” type II amphiphilic helix (Gkeka and Sarkisov, 2010), which plunges into the lipid bilayer parallel to the membrane surface (Figure 2B). Helix 1 of Cr-FAX1 and Cr-FAX5 in this model instead represent type III amphiphilic helices where hydrophilic residues are clustered only at the N-terminal end and thereby the membrane-spanning helix is tilted vertically inside the membrane [compare Könnel et al. (2019)]. 2) In comparison to hm-TMEM14A, however, the first α-helix of Cr-FAX1/FAX5 would be perpendicular to the lipid bilayer membrane, whereas helix 3 and 4 would be tilted vertically (Figure 2C). Because both structural models appear with highest confidence (99.7–99.9% by Phyre^2^; Kelley et al., 2015), we cannot assign the final membrane topology for FAX1 and FAX5/6 proteins, however, in both models helices 1 and 3 display an amphiphilic character.

In addition to Cr-FAX1 and Cr-FAX5 that have been described simplistically earlier [*see* Li et al. (2019)], we could identify two more FAX proteins in Chlamydomonas, i.e. Cr-FAX3 and Cr-FAX7 (Table 1 and Supplementary Figure S2). Cr-FAX3 is most similar to At-FAX3 and, in addition to a cleavable, N-terminal chloroplast transit peptide, has a poly-glycine region N-terminally of the Tmemb_14 domain, which is typical for all plant FAX3 proteins (Supplementary Figure S2A). The mature Cr-FAX3 has a predicted mass of 15.7 kDa. The protein encoded by Cre09.g387838 has about 11.2 kDa and is related to At-FAX7 (Table 1 and Supplementary Figure S2) and therefore was named Cr-FAX7. Like for At-FAX7, no targeting peptide for subcellular localization could be predicted for Cr-FAX7 (Table 1).

Since Cr-FAX1 and Cr-FAX5 have very similar structural features and appear to represent a basic set of chloroplast (Cr-FAX1) and ER (Cr-FAX5) predicted FAX proteins, which is conserved throughout the plant kingdom [compare also Könnel et al. (2019)], we chose to further characterize these two proteins in Chlamydomonas.

### Cr-FAX1 is in chloroplast membranes and Cr-FAX5 in ER membranes of Chlamydomonas cells

**FIGURE 3:**
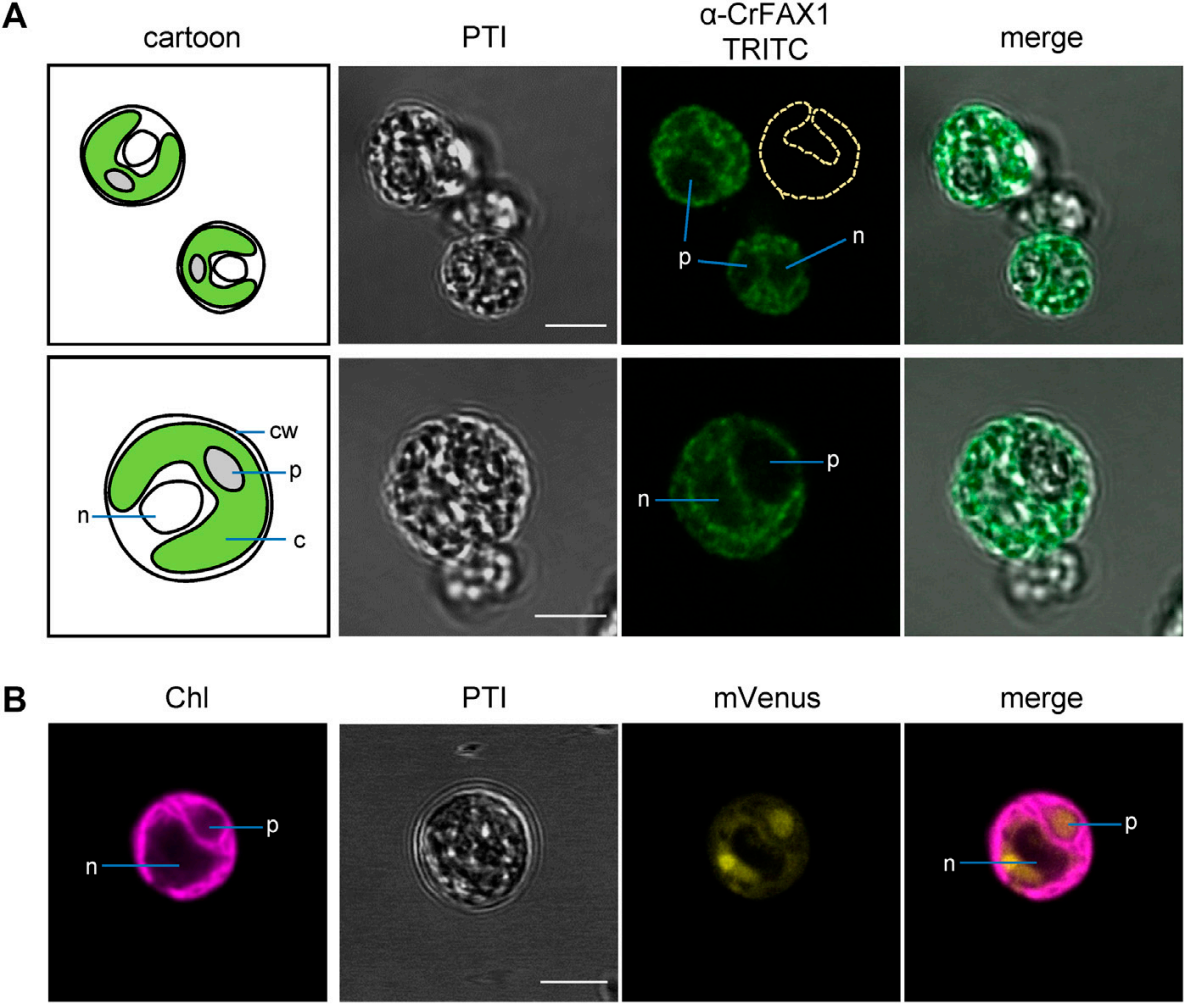
Cr-FAX1 can be found in the chloroplast envelope of Chlamydomonas. **(A)** *In situ* immunofluorescence signals of α-CrFAX1 in Chlamydomonas cells. Intact, fixed wild-type UVM4 *C. reinhardtii* cells were treated with α-CrFAX1 antiserum and signals of the secondary antibody were detected by coupled fluorescence of TRITC (tetramethylrhodamine-isothiocyanate). Shown are pseudo transmission images (PTI), pictures of TRITC-fluorescence (green) and an overlay of both. A cartoon (left) illustrates positioning and organelles of the respective Chlamydomonas cells that were recorded prior to antiserum treatment. Location of the chloroplast envelope and its lobes is indicated by a yellow dashed line (α-CrFAX1/TRITC upper panel). TRITC fluorescence was excited at 561 nm and recorded between 565 and 583 nm by a Leica LSM780 confocal microscope. For a background control of unspecific TRITC fluorescence in comparison to chlorophyll, see Supplementary Figure S4A. c, chloroplast; cw, cell wall; n, nucleus; p, pyrenoid. **(B)** Fluorescence signals of chloroplast controls. Intact, fixed *C. reinhardtii* cells of an UVM4 strain transformed with an mVenus-fluorescence construct, which is targeted to the chloroplast stroma, were examined by fluorescence microscopy. Shown are pseudo transmission images (PTI) as well as chlorophyll (purple) and mVenus (yellow) fluorescence and an overlay of both. (mVenus: excitation at 514 nm, emission at 520–564 nm; chlorophyll: excitation at 458 nm, emission at 626–735 nm; Leica LSM780 confocal microscope). Note that the soluble mVenus construct in **(B)** shows signals in the chloroplast stroma and the pyrenoid, while chlorophyll fluorescence **(B)** and α-CrFAX1 signals in **(A)** only appear at thylakoid and envelope membranes, respectively. All scale bars are 5 μm.

**FIGURE 4:**
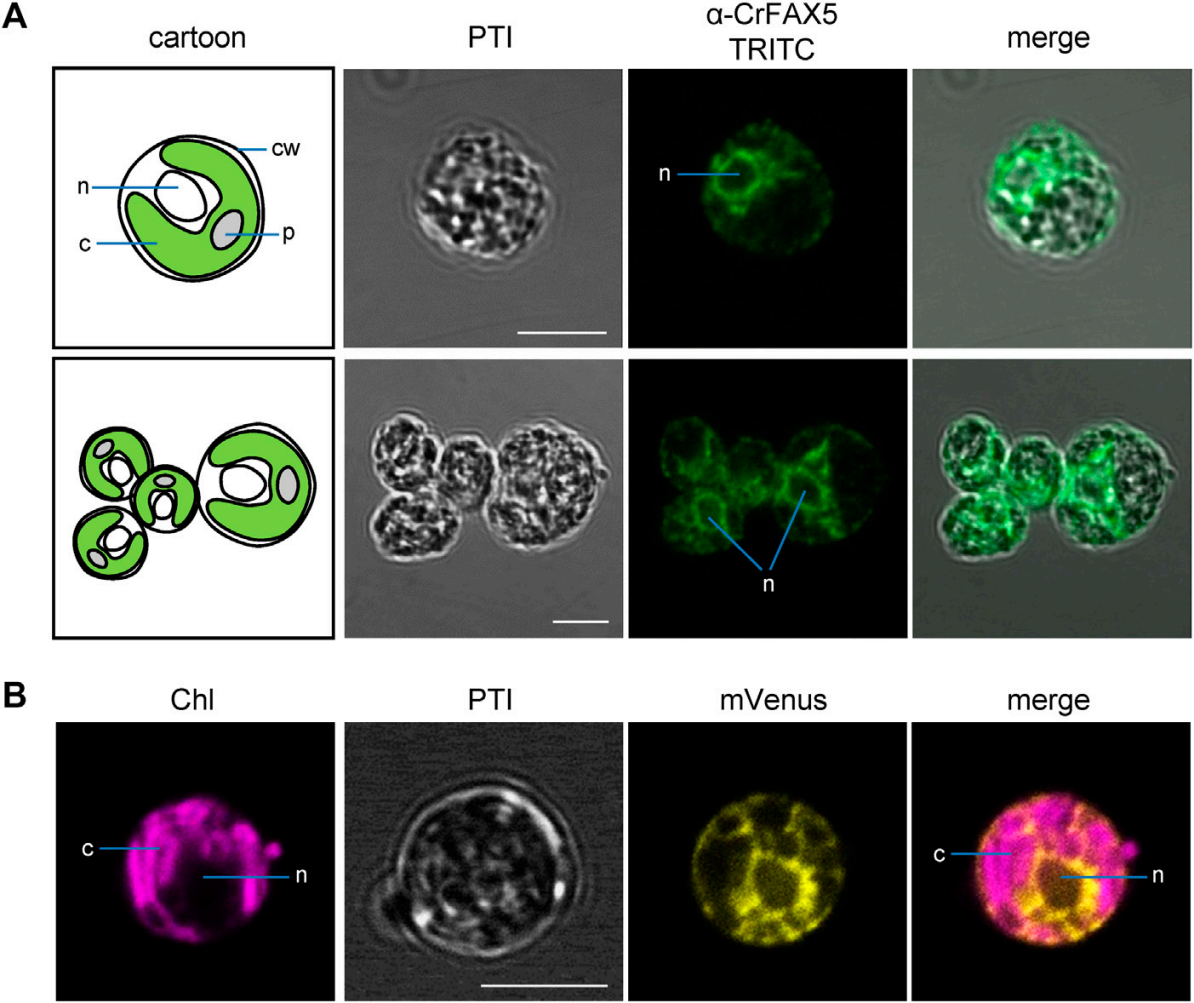
Cr-FAX5 can be found in ER membranes of Chlamydomonas. **(A)** *In situ* immunofluorescence signals of α-CrFAX5 in Chlamydomonas cells. Intact, fixed wild-type UVM4 *C. reinhardtii* cells were treated with α-CrFAX5 antiserum and signals of the secondary antibody were detected by coupled fluorescence of TRITC (tetramethylrhodamine-isothiocyanate). Shown are pseudo transmission images (PTI), pictures of TRITC-fluorescence (green) and an overlay of both. A cartoon (left) illustrates positioning and organelles of the respective Chlamydomonas cells that were recorded prior to antiserum treatment. TRITC fluorescence was excited at 561 nm and recorded between 565 and 583 nm by a Leica LSM780 confocal microscope. For a background control of unspecific TRITC fluorescence in comparison to chlorophyll, see Supplementary Figure S4A. c, chloroplast; cw, cell wall; n, nucleus; p, pyrenoid. **(B)** Fluorescence signals of an ER lumen control. Intact, fixed *C. reinhardtii* cells of an UVM4 strain transformed with an mVenus-fluorescence construct, which is targeted to and retained in the ER lumen, were examined by fluorescence microscopy. Shown are pseudo transmission images (PTI) as well as chlorophyll (purple) and mVenus (yellow) fluorescence and an overlay of both (mVenus: excitation at 514 nm, emission at 520–564 nm; chlorophyll: excitation at 458 nm, emission at 626–735 nm; Leica LSM780 confocal microscope). Note that the signals of mVenus in the ER lumen **(B)** and of α-CrFAX5 **(A)** are very similar surrounding the nucleus and do neither overlap with chloroplasts or chlorophyll fluorescence. All scale bars are 5 μm.

To experimentally verify the subcellular localization of Cr-FAX1 and Cr-FAX5 proteins in Chlamydomonas cells, we generated antisera against C-terminal peptides of both proteins (Figure 2A). Subsequently, we used our antisera, specific for Cr-FAX1 and Cr-FAX5, for immunohistochemical staining. After incubation with a fluorescent secondary antibody, we thereby could follow *in situ* localization in wild-type Chlamydomonas cells. For Cr-FAX1, we could detect specific fluorescent signals in the chloroplast envelope (Figure 3). Compared to respective controls in the literature, Cr-FAX1 has a rather non-homogeneous distribution in the chloroplast envelope, similar to the protein import translocon protein Tic20 [*see* Mackinder et al. (2017)]. Cr-FAX5 signals, however, clearly associated with non-chloroplast membranes connected to the nucleus (Figure 4). In comparison to fluorescent signals of an ER targeted mVenus control (Figure 4B) and to the ER lumen protein BiP (Mackinder et al., 2017), we deduce that these membranes belong to the ER.

Testing of our antisera by immunoblot analysis on proteins from a crude membrane preparation of wild-type Chlamydomonas cells confirms the predicted molecular mass of mature Cr-FAX1 (about 14 kDa) and of Cr-FAX5 (around 11–12 kDa; Supplementary Figure S3A). Moreover, peptide sequencing of a respective SDS gel slice at 14 kDa, exactly validated the predicted processing site of a chloroplast stromal peptidase (compare Figure 2A). Thus, we can conclude that Cr-FAX1 has a cleavable, N-terminal transit peptide of 54 aa and that the mature, processed Cr-FAX1 protein of 14.3 kDa is 145 amino acids long. Because signals of Cr-FAX5 antisera appeared at approximately 11–12 kDa (Supplementary Figure S3A), we assume that the predicted N-terminal, α-helical ER signal-sequence is actually a signal anchor sequence that is not cleaved, as observed for type II integral membrane proteins in the ER (Liaci and Forster, 2021). Indeed, with 19 aa, the first hydrophobic α-helix of Cr-FAX5 is a bit too long to act as cleavable signal peptide. Furthermore, the required small, hydrophobic residues at position -1 and -3 relative to the predicted protease cleavage site are not exactly positioned in Cr-FAX5 (compare with Figure 2A). We thus conclude that the full Cr-FAX5 protein is 121 amino acids long and has a molecular mass around 12 kDa (*see* Table 1). As expected, the signal of α-Cr-FAX1 was highly enriched in fractionated membrane proteins, which in Chlamydomonas cells primarily consist of chloroplast integral membrane proteins as documented by the control Lhcb4 (Supplementary Figure S3A). Remarkably, signals for Cr-FAX5 appeared in the supernatant and not in the pellet of the crude membrane preparation, indicating that this protein most likely localizes to non-chloroplast, light membrane fractions. To further follow Cr-FAX1 and Cr-FAX5 localization, we separated Chlamydomonas chloroplasts and microsomes from other organellar membranes by differential centrifugation, which in particular is described for purification of microsomal membranes. After immunoblot analysis, we here again observed that Cr-FAX1 is in chloroplast membranes. Cr-FAX5 in contrast to Cr-FAX1 and well in line with the *in situ* immunolocalization, clearly associated with the microsomal fraction and co-localized with signals of antisera against the ER-membrane integral Cr-ABCA2 (Jang et al., 2020) and BiP (binding immunoglobulin protein), a marker for the ER lumen (Supplementary Figure S3B).

In summary, we could show by two direct immunological approaches on purified membrane proteins and *in situ* on intact Chlamydomonas wild-type cells, that Cr-FAX1 integrates into the chloroplast envelope and that Cr-FAX5 is an integral membrane protein of the ER. Thus, the mature Cr-FAX1 inserts with its four hydrophobic α-helical domains into a chloroplast envelope membrane, which due to the presence of a classical N-terminal chloroplast transit peptide and in comparison to Arabidopsis and pea FAX1 (Li et al., 2015), most likely is the chloroplast IE. Cr-FAX5 appears to be a type II integral membrane protein of the ER, which contains a signal anchor sequence in the first hydrophobic α-helix.

### The oil content can be manipulated by varying Cr-FAX1 or Cr-FAX5 protein levels

**FIGURE 5:**
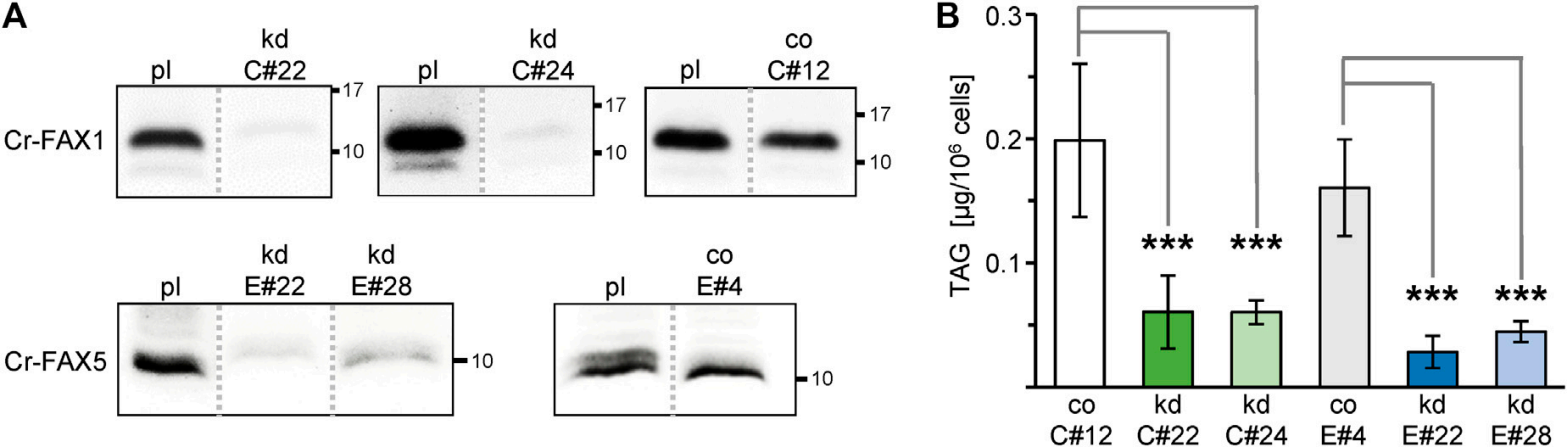
Knockdown of Cr-FAX1 and Cr-FAX5 affects oil content in Chlamydomonas. **(A)** Knockdown of Cr-FAX1 and Cr-FAX5 in cw15-325 Chlamydomonas cells. Immunoblot analysis of Cr-FAX1 and Cr-FAX5 in protein extracts from Chlamydomonas cells that were transformed with amiRNA knockdown constructs for Cr-FAX1 (kd-C, upper panels) and for Cr-FAX5 (kd-E, lower panels) under control of the inducible *NIT1* promoter (see Figure 1A). Proteins were extracted from cells of the independently derived Cr-FAX1 strains kd-C#22, kd-C#24, co-C#12 and Cr-FAX5 strains kd-E#28, kd-E#22, co-E#4 as well as the respective untransformed parental strain cw15-325 (pl). Equal amounts of proteins (isolated from the same quantity of cells corresponding to 2 μg chlorophyll) were separated by SDS-PAGE and subjected to immunoblot analysis using antisera directed against Cr-FAX1 (upper panels) and Cr-FAX5 (lower panels). Numbers indicate molecular mass of proteins in kDa. Note that all protein samples were extracted from non-induced Chlamydomonas cells, i.e., knockdowns result from leaky expression of the *NIT1* promoter. While for the strains kd-C#22, kd-C#24, and kd-E#22, kd-E#28, we observed a strong reduction of Cr-FAX1 and Cr-FAX5 proteins, respectively, the strains co-C#12, co-E#4 showed no knockdown effect under these conditions and thus were used as control (co) lines. All boxed immunoblot signals were generated on one identical blot with equal amounts of sample loaded. Signals of other strains, which are not subject of this study, have been removed as indicated by gray, dotted lines (compare Supplementary Figure S5). **(B)** Knockdown (kd) of chloroplast Cr-FAX1 and ER-localized Cr-FAX5 decreases TAG oil content in Chlamydomonas cells. Two independently generated *C. reinhardtii* strains each for knockdown of Cr-FAX1: lines kd-C#22 (green) and kd-C#24 (light green), and Cr-FAX5: lines kd-E#22 (blue) and kd-E#28 (light blue) as well as the respective control lines with wild-type levels for Cr-FAX1 (co-C#12; white) and Cr-FAX5 (co-E#4; gray) were grown in TAP medium and harvested in the exponential growth phase for determination of neutral lipids via thin layer chromatography (TLC). For protein levels in all lines see **(A)**. TAG neutral lipids [μg/10^6^ cells] were quantified densitometrically after separation by TLC from five individual liquid cultures of each strain (n = 5 ± SD). *p*-values for significantly different TAG content when compared to the respective control strains (co-C#12 for Cr-FAX1kd, co-E#4 for Cr-FAX5kd) are indicated (double-sided student t-test): *** *p* < 0.001.

To dissect the function of the two FAX proteins in lipid homeostasis, we generated mutant lines using an artificial microRNA (amiRNA) approach (Figure 1A). Two independent lines for each gene were validated as true and constitutive knockdown of Cr-FAX1 or Cr-FAX5, respectively as shown by immunoblot (Figure 5A). In addition, these immunoblots verified the specificity of our antisera α-CrFAX1 and α-CrFAX5 (compare Supplementary Figure S3) as well as the apparent molecular mass of the mature Cr-FAX1 and Cr-FAX5 proteins (*see* above and Table 1). Although under control of the inducible *NIT1* (nitrate reductase 1) promoter (Schmollinger et al., 2010), the strains kd-C#22, kd-C#24 for Cr-FAX1 and kd-E#22, kd-E#28 for Cr-FAX5 (Figure 5A and Supplementary Figure S5) constitutively showed a strong reduction of FAX proteins. This constitutive activity of the *NIT1* promoter in some transformants most likely is due to position effects imposed by the chromatin structure at the ectopic integration site of the transgene in the genome (Schroda, 2019). The strains C#12 and E#4, however, without *NIT1* induction did not exhibit reduced Cr-FAX1 and Cr-FAX5 protein levels, respectively (compare Supplementary Figure S5). Therefore, we used these two lines as controls with wild type-like Cr-FAX1 and Cr-FAX5 protein content under non-inducing growth conditions. Lipid analysis of exponentially grown cells showed that all four Cr-FAX knockdown strains made around 60% less TAG than their corresponding control lines (Figure 5B). Among all membrane lipids, only DGDG and DGTS (diacylglyceryl-trimethyl-homoserine) showed significant reduction by 15 and 45%, respectively, in the Cr-FAX5 knockdown lines (Supplementary Figure S6A). Knockdown of Cr-FAX1, however, did not result in any variations in content of polar lipid classes. Further, the total lipid content and the distribution of different FA species in all lipids analyzed did not change in any of the Cr-FAX1, Cr-FAX5 knockdown strains when compared to the respective controls (Supplementary Figures 6B,C).

**FIGURE 6:**
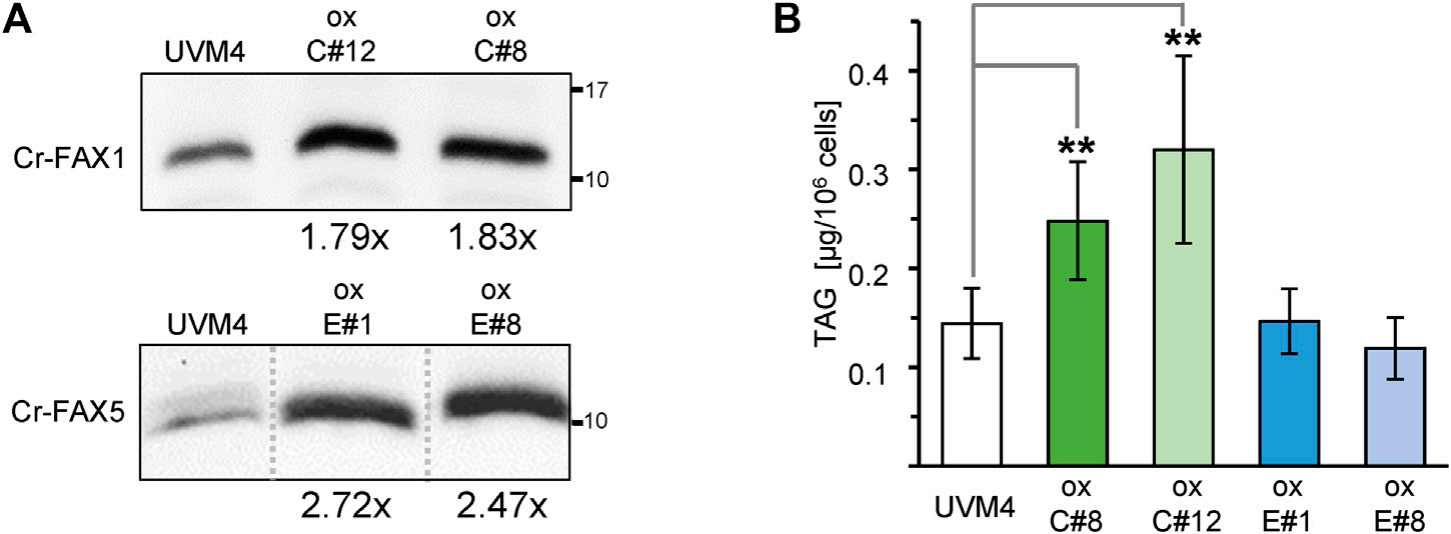
Overexpression of Cr-FAX1 affects oil content in Chlamydomonas. **(A)** Overexpression of Cr-FAX1, Cr-FAX5 in UVM4 *C. reinhardtii* cells. Immunoblot analysis of Cr-FAX1 (top) and Cr-FAX5 (bottom) in protein extracts from Chlamydomonas cells that were transformed with overexpression constructs for Cr-FAX1 (ox-C) and Cr-FAX5 (ox-E) under control of the constitutive *PSAD* promoter (see Figure 1B). Proteins were extracted from cells of independently derived Cr-FAX1ox strains C#12, C#8 (top) and Cr-FAX5ox strains E#1, E#8 (bottom) as well as the respective untransformed, parental strain UVM4. Equal amounts of proteins (isolated from the same quantity of cells corresponding to 2 μg chlorophyll) were separated by SDS-PAGE and subjected to immunoblot analysis using antisera directed against Cr-FAX1 or Cr-FAX5. Numbers indicate molecular mass of proteins in kDa. The increase in protein amount compared to UVM4 could be estimated to be around 1.8-fold for both Cr-FAX1ox C#8 (1.83 ± 0.13), C#12 (1.79 ± 0.16) and at 2.7-fold, 2.5-fold for Cr-FAX5ox E#1 (2.72 ± 0.38) and E#8 (2.47 ± 0.07), respectively (n = 3–4 independent immunoblots ± SD). Note that all boxed immunoblot signals were generated on one identical blot (see Supplementary Table S7). Signals of other mutant lines, which are not subject of this study, have been removed as indicated by gray, dotted lines. **(B)** Overexpression of chloroplast Cr-FAX1 increases TAG oil content in Chlamydomonas cells. Two independently generated *C. reinhardtii* strains (see **(A)**) each for overexpression of Cr-FAX1: lines ox-C#8 (green) and ox-C#12 (light green), and Cr-FAX5: lines ox-E#1 (blue) and ox-E#8 (light blue) as well as the respective wild-type parental strain UVM4 (white) were grown in TAP medium and harvested in the exponential growth phase for determination of neutral lipids via TLC. TAG neutral lipids [μg/10^6^ cells] were quantified densitometrically after separation by TLC from five individual liquid cultures of each strain (n = 5 ± SD). *p*-values for significantly different TAG contents when compared to UVM4 are indicated (double-sided student t-test): ** *p* < 0.01.

To further elucidate the impact of Cr-FAX proteins on lipid homeostasis in Chlamydomonas, we generated overexpression lines for each gene *Cr-FAX1* and *Cr-FAX5* (Figure 1B) under control of the constitutive and strong promoter for photosystem I reaction center subunit II (*PSAD*; Fischer and Rochaix, 2001). Overexpression in two independent strains for each Cr-FAX1 (ox-C#8, ox-C#12) and Cr-FAX5 (ox-E#1, ox-E#8) was confirmed by immunoblotting (Figure 6A and Supplementary Figure S7). Lipid analysis in these lines revealed that overexpression of Cr-FAX1 in Chlamydomonas almost doubled the TAG content when compared to the parental strain UVM4 (Figure 6B). Overexpression of Cr-FAX5, however, did not bring any difference in TAG levels, but showed a mild reduction in MGDG and DGDG polar lipids (Supplementary Figure S8A). Similar to the knockdown lines, the total lipid content in all overexpression strains did not substantially change in comparison to the UVM4 parental line (Supplementary Figure S8B). However, some modifications could be observed in the distribution of FA molecule species (Supplementary Figure S8C): most prominent was a reduction of C16:1(7) and the C18:1(9) FAs in lipids from all overexpression strains for Cr-FAX1 or Cr-FAX5, respectively.

In summary, knockdown of both Cr-FAX1 and Cr-FAX5 led to a strong decrease in TAG levels in Chlamydomonas, whereas overexpression only of the chloroplast Cr-FAX1 almost doubled the TAG content. Effects on polar lipids and FA species in acyl lipids, however, were only marginal. Since all mutant strains grew normally under standard cultivation conditions (Supplementary Figure S9), we conclude that changes in TAG content are a direct effect of changing Cr-FAX protein levels.

## Discussion

**FIGURE 7:**
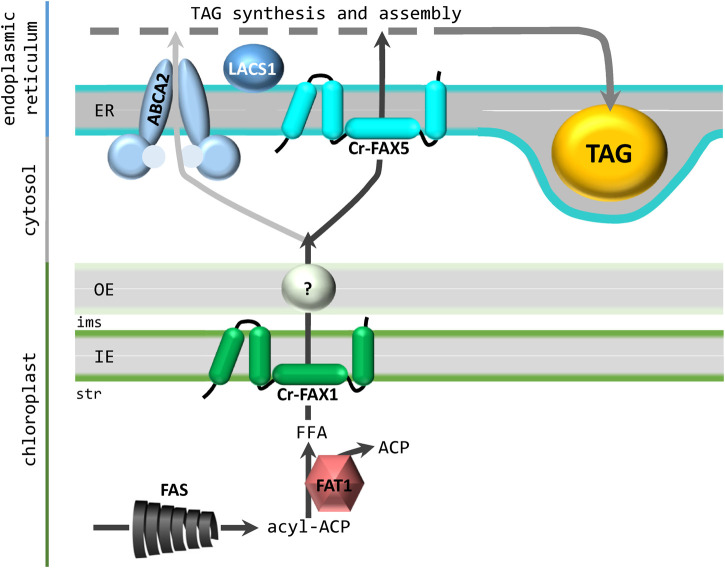
Contribution of Cr-FAX1 and Cr-FAX5 for oil synthesis in Chlamydomonas. According to our working hypothesis, Cr-FAX1 with its 4 α-helical domains (green) integrates in an asymmetric fashion into the inner envelope (IE) membrane of chloroplasts. Most likely the N-terminus is in the stroma (str) and the C-terminus in the intermembrane space (ims). In comparison to proteolytic pattern of Ps-FAX1 in purified IE membrane vesicles from pea chloroplasts (JP and KP, unpublished results), Cr-FAX1 presumably orients with the N-terminus to the stroma and the C-terminus exposed to the ims. Upon proteolysis of this C-terminus of Ps-FAX1, intermediate bands can be detected by an antiserum directed against an N-terminal peptide stretch (JP and KP, unpublished results). Since the C-terminus of the structural model shown in Figure 2C is almost completely protected by the lipid bilayer, the model of Figure 2B, which is adapted here, is more likely to lead to these intermediate band pattern. Cr-FAX5 (turquoise) in a similar fashion sits in the membrane of the endoplasmic reticulum (ER). De novo fatty acid synthesis (FAS) occurs in the chloroplast stroma and the acyl-ACP thioesterase 1 (FAT1) generates free fatty acids (FFA) for export mediated by FAX1. The transport mode for FAs across the outer envelope (OE) membrane still is unclear. Import of FFAs and/or acyl-CoA into the ER occurs via the ABC transporter ABCA2 and with the help of FAX5. If both proteins act together or represent different FA import routes remains to be elucidated. However, function of ABCA2 and FAX5, most likely contributes acyl chains for triacylglycerol (TAG) synthesis and assembly in the ER. As described in the text, we assume that FAT1, FAX1, FAX5 and LACS1 (long chain acyl CoA synthetase 1) function in the same transport pathway from chloroplast to the ER for fueling FAs into TAG oil synthesis.

In this study, we provide a general overview on FAX proteins in the green microalga *Chlamydomonas reinhardtii* in comparison to the model plant *Arabidopsis thaliana*. With Cr-FAX1/Cr-FAX5 and, most likely, also with Cr-FAX3/Cr-FAX7, Chlamydomonas appears to harbor two basic sets of FAX proteins, in which one FAX is targeted to membranes of chloroplasts (FAX1 and FAX3) and the other to the ER/secretory pathway (FAX5 and FAX7). Members of FAX2 and FAX4 subfamilies, which can be found in land plants, appear to be absent in the Chlamydomonas genome. Due to similar secondary structure and conserved amino acid sequence motifs of Cr-FAX1 and Cr-FAX5, an analogous function of both membrane proteins is likely. Since Cr-FAX1 is predicted to be localized to the chloroplast envelope and Cr-FAX5 to ER/secretory pathway membranes, we chose to verify their subcellular localization and to investigate their function. In contrast to a previous report, where the protein equivalent to Cr-FAX5 was assumed to be in chloroplasts (Li et al., 2019), here by *in situ* immunolocalization we show unequivocally that Cr-FAX5 in Chlamydomonas cells is targeted to ER membranes. Although an N-terminal ER signal sequence is weakly predicted for Cr-FAX5, the protein of around 12 kDa (121 amino acids) most likely is not processed and represents a type II integral membrane protein of the ER (Liaci and Forster, 2021). Further, we provide experimental evidence that Cr-FAX1 inserts into the chloroplast envelope. Due to determination of the processing site for a chloroplast stromal peptidase by peptide sequencing, we conclude that the mature Cr-FAX1 protein contains 145 amino acids with a molecular mass of about 14.3 kDa. Whereas in the unicellular red alga *C. merolae*, chloroplast envelope localization of the FAX1 ortholog Cm-FAX1 was demonstrated on overexpression strains with FLAG-tagged Cm-FAX1 (Takemura et al., 2019), our studies rely on endogenous protein levels in non-modified UVM4 cells, detected by specific antisera for Cr-FAX1 and Cr-FAX5. In comparison to the IE-membrane intrinsic At-FAX1 and Ps-FAX1 (Li et al., 2015), Cr-FAX1 with its four α-helical domains most likely integrates into the IE membrane of Chlamydomonas chloroplasts as well. In comparison to proteolytic pattern of Ps-FAX1 in purified IE membrane vesicles from pea chloroplasts (JP and KP, unpublished results), Cr-FAX1 presumably orients with the N-terminus to the stroma and the C-terminus exposed to the inter membrane space (Figure 7). Because in Chlamydomonas as well as in Arabidopsis [compare Könnel et al. (2019)], secondary structure and the predicted membrane topology of mature FAX1 and FAX5 proteins are very similar, we suppose that Cr-FAX1 and Cr-FAX5 function in the same metabolic pathway in chloroplast envelope and ER membranes, respectively.

To test this assumption, we generated amiRNA knockdown strains for Cr-FAX1 and Cr-FAX5 in Chlamydomonas, resulting in a drastic reduction of FAX protein levels. In land plants, the function of plastid-intrinsic FAX-proteins is associated with a role in export of FAs from plastids and hence an effect on the homeostasis of lipid compounds throughout plant development (Li et al., 2015; Tian et al., 2018; Tian et al., 2019; Li et al., 2020; Zhu et al., 2020; Cai et al., 2021; Huang et al., 2021; Xiao et al., 2021). The *in planta* task of ER-localized FAX5/6, to our knowledge, has not been elucidated yet. In consequence, we here studied the impact of reduced levels of Cr-FAX1 and Cr-FAX5 proteins on lipid homeostasis in Chlamydomonas. Remarkably, knockdown lines for both Cr-FAX1 and Cr-FAX5 exhibited a strong and reproducible decrease in ER-made TAG, with levels down to 30% of controls in both Cr-FAX1 knockdowns, and to 18 and 28% in the two Cr-FAX5 knockdowns. The total lipid content and distribution of FA-molecule species in acyl lipids did not change and polar lipids were only slightly affected in Cr-FAX5 knockdowns, indicating that potential membrane lipid remodeling (Young and Shachar-Hill, 2021) to compensate for the reduction in TAG does not occur. However, the pronounced reduction of the ER-produced DGTS to 46 and 62.5% in both Cr-FAX5 knockdown lines points to an ER-specific function of Cr-FAX5 in lipid synthesis. Thus, in Chlamydomonas we can provide a proof of principle for the implication of chloroplast FAX1 proteins in TAG production and show that a FAX5 protein in the ER membrane is involved in the same physiological process, most likely the transfer of FAs from chloroplasts into the ER for assembly of acyl lipids (Figure 7).

In comparison to the Chlamydomonas ER membrane-intrinsic ABC-transporter CrABCA2, which most likely imports FAs and/or acyl-CoA into the ER and has been described to be crucial for TAG accumulation under nitrogen deprivation (Jang et al., 2020), the contribution of Cr-FAX1 and Cr-FAX5 to TAG assembly appears to be stronger. Knockdowns of CrABCA2 only reduced TAG levels to 70–80% of the corresponding wild type, although values are difficult to compare because of different Chlamydomonas strains, promoters, and growth conditions. Knockdowns for Cr-FAX1 have not been described in the literature, however, a knockout strain of the *C. merolae* ortholog Cm-FAX1 did not affect red algal TAG levels (Takemura et al., 2019). In Arabidopsis, the loss of At-FAX1 function leads to a decrease in TAG content by only 4.3–7.2% in leaf and flower tissue, respectively. Thus, the effect of reduced chloroplast Cr-FAX1 protein levels on TAG production in Chlamydomonas appears to be more stringent and direct than in *C. merolae* and Arabidopsis. In Arabidopsis, redundancy of plastid IE- localized FAX proteins – i.e. FAX1, FAX2, FAX3, FAX4 – might compensate for the loss of FAX1 in single knockouts, while in Chlamydomonas only the chloroplast predicted Cr-FAX3 could functionally replace Cr-FAX1.

To further track the function of FAX proteins in lipid homeostasis and to contribute knowledge for potential biofuel production in microalgae, we also generated overexpression strains for Cr-FAX1 and Cr-FAX5. While the amiRNA generated knockdown of Cr-FAX1 and Cr-FAX5 was quite efficient, overexpression under control of the constitutive *PSAD* promoter in comparison was rather modest with about 1.8-fold and 2.5–2.7-fold higher protein levels for Cr-FAX1 and Cr-FAX5, respectively. However, this mild increase in Cr-FAX1 protein levels resulted in a doubling of the oil content: line Cr-FAX1ox C#8 produced 1.7-fold, and line Cr-FAX1ox C#12 produced 2.2-fold more TAG when compared to their parental strain. In contrast, increase of Cr-FAX5 proteins did not affect TAG levels. Similar to knockdown lines, changes in total lipid content could not be recorded and the polar lipids MGDG and DGDG were only marginally reduced in Cr-FAX5 overexpression lines. Most prominent for the distribution of FA species in total lipids was a reduction of C16:1(7) and C18:1(9) FAs in lipids of all overexpression strains for Cr-FAX1 or Cr-FAX5. Both FA species are desaturated within the chloroplast, and their reduction in relative proportion in FAX overexpressing lines suggests that an enhanced flow of FAs between subcellular organelles can affect FA desaturation levels. Our results for Cr-FAX1 overexpression are comparable to the very strong overexpression of Cm-FAX1, which leads to a 2.4-fold increase in total TAG in *C. merolae* cells (Takemura et al., 2019). The reported 1.3-fold increase of TAG in single lines supposedly overexpressing Cr-FAX1 and Cr-FAX5 in Chlamydomonas (Li et al., 2019) are difficult to relate, because the only very marginal rise of transcripts by around 1.5- and 1.3-fold for CrFAX1 and CrFAX2/alias Cr-FAX5, respectively, casts doubts on the overexpression at the protein level. Also, the strong changes in polar lipids, observed by Li and coworkers (2019) in their strains cannot be reproduced by our study. In Arabidopsis, very strong overexpression of At-FAX1 results in a quite modest increase of TAG content when compared to wild type tissue, i.e. 3.2% in leaf and 6.6% in flower tissue (Li et al., 2015). Thus, the impact of moderately increased protein levels of chloroplast IE-intrinsic FAX1 in Chlamydomonas is significant, and the protein definitely represents a target for biotechnological oil production in unicellular green and red microalgae like *Chlamydomonas reinhardtii* and *Cyanidioshyzon merolae.*

Interestingly, in contrast to overexpression of Cr-FAX1, overexpression of Cr-FAX5 in our study did not affect the TAG content in Chlamydomonas cells. In strains with increased levels of the ER intrinsic FA/acyl-CoA importer CrABCA2, however, the TAG yield could be mildly improved by 1.2–1.6-fold under standard conditions (Jang et al., 2020). It is thus tempting to speculate that a bottleneck for increasing Chlamydomonas oil content might rather be in FA export from chloroplasts *via* FAX1 proteins than in FA/acyl-CoA import into the ER, mediated by FAX5. Only very recently, *Cr-FAX1* and *Cr-FAX5* were described to be co-expressed with chloroplast-intrinsic acyl-ACP thioesterase 1 (*FAT1*), and the long-chain acyl CoA synthetase *LACS1*, indicating that these genes function in the same pathway for enhanced FA transport from chloroplast to ER for TAG production (Choi et al., 2022). Well in line with a chloroplast FA-delivery bottleneck for TAG assembly in the ER, overexpression of *Cr-FAT1*, which generates free fatty acids for export into the chloroplast stroma by Choi and coworkers (2022) could increase the TAG content by 1.5-fold.

In summary, we conclude that Cr-FAX1 in the chloroplast IE and Cr-FAX5 in the ER membrane function together in shuttling FA acyl chains from the site of synthesis in the chloroplast stroma to the ER lumen for TAG lipid assembly (Figure 7).

## Data Availability

The original contributions presented in the study are included in the article/Supplementary Material, further inquiries can be directed to the corresponding author.

## References

[B1] Almagro Armenteros J. J. Salvatore M. Emanuelsson O. Winther O. von Heijne G. Elofsson A. (2019). Detecting sequence signals in targeting peptides using deep learning. Life Sci. Alliance 2, e201900429. 10.26508/lsa.201900429 31570514 PMC6769257

[B2] Bates P. D. (2016). Understanding the control of acyl flux through the lipid metabolic network of plant oil biosynthesis. Biochim. Biophys. Acta 1861, 1214–1225. 10.1016/j.bbalip.2016.03.021 27003249

[B3] Blatti J. L. Michaud J. Burkart M. D. (2013). Engineering fatty acid biosynthesis in microalgae for sustainable biodiesel. Curr. Opin. Chem. Biol. 17, 496–505. 10.1016/j.cbpa.2013.04.007 23683348

[B4] Block M. A. Jouhet J. (2015). Lipid trafficking at endoplasmic reticulum-chloroplast membrane contact sites. Curr. Opin. Cell Biol. 35, 21–29. 10.1016/j.ceb.2015.03.004 25868077

[B6] Cai G. Wang G. Kim S. C. Li J. Zhou Y. Wang X. (2021). Increased expression of fatty acid and ABC transporters enhances seed oil production in camelina. Biotechnol. Biofuels 14, 49. 10.1186/s13068-021-01899-w 33640013 PMC7913393

[B7] Choi B. Y. Shim D. Kong F. Auroy P. Lee Y. Li-Beisson Y. (2022). The Chlamydomonas transcription factor MYB1 mediates lipid accumulation under nitrogen depletion. New Phytol. 235, 595–610. 10.1111/nph.18141 35383411

[B8] Crozet P. Navarro F. J. Willmund F. Mehrshahi P. Bakowski K. Lauersen K. J. (2018). Birth of a photosynthetic chassis: a MoClo toolkit enabling synthetic biology in the microalga *Chlamydomonas reinhardtii* . ACS Synth. Biol. 7, 2074–2086. 10.1021/acssynbio.8b00251 30165733

[B9] Duy D. Wanner G. Meda A. R. von Wiren N. Soll J. Philippar K. (2007). PIC1, an ancient permease in Arabidopsis chloroplasts, mediates iron transport. Plant Cell 19, 986–1006. 10.1105/tpc.106.047407 17337631 PMC1867359

[B10] Fischer N. Rochaix J. D. (2001). The flanking regions of PsaD drive efficient gene expression in the nucleus of the green alga *Chlamydomonas reinhardtii* . Mol. Genet. Genomics 265, 888–894. 10.1007/s004380100485 11523806

[B11] Gkeka P. Sarkisov L. (2010). Interactions of phospholipid bilayers with several classes of amphiphilic alpha-helical peptides: insights from coarse-grained molecular dynamics simulations. J. Phys. Chem. B 114, 826–839. 10.1021/jp908320b 20028006

[B12] Goodstein D. M. Shu S. Howson R. Neupane R. Hayes R. D. Fazo J. (2012). Phytozome: a comparative platform for green plant genomics. Nucleic Acids Res. 40, D1178–D1186. 10.1093/nar/gkr944 22110026 PMC3245001

[B13] Gu X. Cao L. Wu X. Li Y. Hu Q. Han D. (2021). A lipid bodies-associated galactosyl hydrolase is involved in triacylglycerol biosynthesis and galactolipid turnover in the unicellular green alga *Chlamydomonas reinhardtii* . Plants (Basel) 10, 675. 10.3390/plants10040675 33807496 PMC8065580

[B14] Huang K. L. Tian J. Wang H. Fu Y. F. Li Y. Zheng Y. (2021). Fatty acid export protein BnFAX6 functions in lipid synthesis and axillary bud growth in Brassica napus. Plant Physiol. 186, 2064–2077. 10.1093/plphys/kiab229 34618109 PMC8331132

[B15] Hurlock A. K. Roston R. L. Wang K. Benning C. (2014). Lipid trafficking in plant cells. Traffic 15, 915–932. 10.1111/tra.12187 24931800

[B16] Jang S. Kong F. Lee J. Choi B. Y. Wang P. Gao P. (2020). CrABCA2 facilitates triacylglycerol accumulation in *Chlamydomonas reinhardtii* under nitrogen starvation. Mol. Cells 43, 48–57. 10.14348/molcells.2019.0262 31910336 PMC6999713

[B17] Kelley L. A. Mezulis S. Yates C. M. Wass M. N. Sternberg M. J. (2015). The Phyre2 web portal for protein modeling, prediction and analysis. Nat. Protoc. 10, 845–858. 10.1038/nprot.2015.053 25950237 PMC5298202

[B18] Kim S. Yamaoka Y. Ono H. Kim H. Shim D. Maeshima M. (2013). AtABCA9 transporter supplies fatty acids for lipid synthesis to the endoplasmic reticulum. Proc. Natl. Acad. Sci. U. S. A. 110, 773–778. 10.1073/pnas.1214159110 23269834 PMC3545803

[B19] Kim Y. Terng E. L. Riekhof W. R. Cahoon E. B. Cerutti H. (2018). Endoplasmic reticulum acyltransferase with prokaryotic substrate preference contributes to triacylglycerol assembly in *Chlamydomonas* . Proc. Natl. Acad. Sci. U. S. A. 115, 1652–1657. 10.1073/pnas.1715922115 29382746 PMC5816170

[B20] Kindle K. L. (1990). High-frequency nuclear transformation of *Chlamydomonas reinhardtii* . Proc. Natl. Acad. Sci. U. S. A. 87, 1228–1232. 10.1073/pnas.87.3.1228 2105499 PMC53444

[B21] Klammt C. Maslennikov I. Bayrhuber M. Eichmann C. Vajpai N. Chiu E. J. (2012). Facile backbone structure determination of human membrane proteins by NMR spectroscopy. Nat. Methods 9, 834–839. 10.1038/nmeth.2033 22609626 PMC3723349

[B22] Könnel A. Bugaeva W. Guegel I. L. Philippar K. (2019). BANFF: bending of bilayer membranes by amphiphilic alpha-helices is necessary for form and function of organelles^1^ . Biochem. Cell Biol. 97, 243–256. 10.1139/bcb-2018-0150 30208283

[B23] Kropat J. Hong-Hermesdorf A. Casero D. Ent P. Castruita M. Pellegrini M. (2011). A revised mineral nutrient supplement increases biomass and growth rate in *Chlamydomonas reinhardtii* . Plant J. 66, 770–780. 10.1111/j.1365-313X.2011.04537.x 21309872 PMC3101321

[B24] LaBrant E. Barnes A. C. Roston R. L. (2018). Lipid transport required to make lipids of photosynthetic membranes. Photosynth. Res. 138, 345–360. 10.1007/s11120-018-0545-5 29961189

[B25] Lavell A. A. Benning C. (2019). Cellular organization and regulation of plant glycerolipid metabolism. Plant Cell Physiol. 60, 1176–1183. 10.1093/pcp/pcz016 30690552 PMC6553661

[B26] Legeret B. Schulz-Raffelt M. Nguyen H. M. Auroy P. Beisson F. Peltier G. (2016). Lipidomic and transcriptomic analyses of *Chlamydomonas reinhardtii* under heat stress unveil a direct route for the conversion of membrane lipids into storage lipids. Plant Cell Environ. 39, 834–847. 10.1111/pce.12656 26477535

[B27] Li N. Gügel I. L. Giavalisco P. Zeisler V. Schreiber L. Soll J. (2015). FAX1, a novel membrane protein mediating plastid fatty acid export. PLoS Biol. 13, e1002053. 10.1371/journal.pbio.1002053 25646734 PMC4344464

[B28] Li N. Xu C. Li-Beisson Y. Philippar K. (2016). Fatty acid and lipid transport in plant cells. Trends Plant Sci. 21, 145–158. 10.1016/j.tplants.2015.10.011 26616197

[B29] Li N. Zhang Y. Meng H. Li S. Wang S. Xiao Z. (2019). Characterization of Fatty Acid Exporters involved in fatty acid transport for oil accumulation in the green alga *Chlamydomonas reinhardtii* . Biotechnol. Biofuels 12, 14. 10.1186/s13068-018-1332-4 30651755 PMC6330502

[B30] Li N. Meng H. Li S. Zhang Z. Zhao X. Wang S. (2020). Two plastid fatty acid exporters contribute to seed oil accumulation in Arabidopsis. Plant Physiol. 182, 1910–1919. 10.1104/pp.19.01344 32019874 PMC7140923

[B31] Liaci A. M. Forster F. (2021). Take me home, protein roads: Structural insights into signal peptide interactions during ER translocation. Int. J. Mol. Sci. 22, 11871. 10.3390/ijms222111871 34769302 PMC8584900

[B32] Li-Beisson Y. Shorrosh B. Beisson F. Andersson M. X. Arondel V. Bates P. D. (2013). “Acyl-lipid metabolism,” in The Arabidopsis book (American Society of Plant Biologists), 11, e0161. 23505340 10.1199/tab.0161 PMC3563272

[B33] Li-Beisson Y. Beisson F. Riekhof W. (2015). Metabolism of acyl-lipids in *Chlamydomonas reinhardtii* . Plant J. 82, 504–522. 10.1111/tpj.12787 25660108

[B34] Li-Beisson Y. Neunzig J. Lee Y. Philippar K. (2017). Plant membrane-protein mediated intracellular traffic of fatty acids and acyl lipids. Curr. Opin. Plant Biol. 40, 138–146. 10.1016/j.pbi.2017.09.006 28985576

[B35] Li-Beisson Y. Thelen J. J. Fedosejevs E. Harwood J. L. (2019). The lipid biochemistry of eukaryotic algae. Prog. Lipid Res. 74, 31–68. 10.1016/j.plipres.2019.01.003 30703388

[B36] Li-Beisson Y. Kong F. Wang P. Lee Y. Kang B. H. (2021). The disassembly of lipid droplets in *Chlamydomonas* . New Phytol. 231, 1359–1364. 10.1111/nph.17505 34028037

[B37] Mackinder L. C. M. Chen C. Leib R. D. Patena W. Blum S. R. Rodman M. (2017). A spatial interactome reveals the protein organization of the algal CO2-concentrating mechanism. Cell 171, 133–147.e14. 10.1016/j.cell.2017.08.044 28938113 PMC5616186

[B38] Merchant S. S. Kropat J. Liu B. Shaw J. Warakanont J. (2012). TAG, you're it! Chlamydomonas as a reference organism for understanding algal triacylglycerol accumulation. Curr. Opin. Biotechnol. 23, 352–363. 10.1016/j.copbio.2011.12.001 22209109

[B39] Michaud M. Jouhet J. (2019). Lipid trafficking at membrane contact sites during plant development and stress response. Front. Plant Sci. 10, 2. 10.3389/fpls.2019.00002 30713540 PMC6346683

[B40] Molnar A. Bassett A. Thuenemann E. Schwach F. Karkare S. Ossowski S. (2009). Highly specific gene silencing by artificial microRNAs in the unicellular alga *Chlamydomonas reinhardtii* . Plant J. 58, 165–174. 10.1111/j.1365-313X.2008.03767.x 19054357

[B41] Neupert J. Karcher D. Bock R. (2009). Generation of Chlamydomonas strains that efficiently express nuclear transgenes. Plant J. 57, 1140–1150. 10.1111/j.1365-313X.2008.03746.x 19036032

[B42] Ossowski S. Schwab R. Weigel D. (2008). Gene silencing in plants using artificial microRNAs and other small RNAs. Plant J. 53, 674–690. 10.1111/j.1365-313X.2007.03328.x 18269576

[B43] Patron N. J. Orzaez D. Marillonnet S. Warzecha H. Matthewman C. Youles M. (2015). Standards for plant synthetic biology: a common syntax for exchange of DNA parts. New Phytol. 208, 13–19. 10.1111/nph.13532 26171760

[B44] Porra R. J. Scheer H. (1989). Towards a more accurate future for chlorophyll a and b determinations: the inaccuracies of daniel arnon's assay. Photosynth. Res. 140, 215–219. 10.1007/s11120-018-0579-8 30194670

[B45] Rütgers M. Muranaka L. S. Mühlhaus T. Sommer F. Thoms S. Schurig J. (2017). Substrates of the chloroplast small heat shock proteins 22E/F point to thermolability as a regulative switch for heat acclimation in *Chlamydomonas reinhardtii* . Plant Mol. Biol. 95, 579–591. 10.1007/s11103-017-0672-y 29094278 PMC5700999

[B46] Schmollinger S. Strenkert D. Schroda M. (2010). An inducible artificial microRNA system for Chlamydomonas reinhardtii confirms a key role for heat shock factor 1 in regulating thermotolerance. Curr. Genet. 56, 383–389. 10.1007/s00294-010-0304-4 20449593

[B47] Schroda M. (2019). Good news for nuclear transgene expression in *Chlamydomonas* . Cells 8, 1534. 10.3390/cells8121534 PMC6952782 31795196

[B48] Schwacke R. Schneider A. van der Graaff E. Fischer K. Catoni E. Desimone M. (2003). ARAMEMNON, a novel database for Arabidopsis integral membrane proteins. Plant Physiol. 131, 16–26. 10.1104/pp.011577 12529511 PMC166783

[B49] Scranton M. A. Ostrand J. T. Fields F. J. Mayfield S. P. (2015). Chlamydomonas as a model for biofuels and bio-products production. Plant J. 82, 523–531. 10.1111/tpj.12780 25641390 PMC5531182

[B50] Spaniol B. Lang J. Venn B. Schake L. Sommer F. Mustas M. (2022). Complexome profiling on the Chlamydomonas lpa2 mutant reveals insights into PSII biogenesis and new PSII associated proteins. J. Exp. Bot. 73, 245–262. 10.1093/jxb/erab390 34436580 PMC8730698

[B51] Takemura T. Imamura S. Tanaka K. (2019). Identification of a chloroplast fatty acid exporter protein, CmFAX1, and triacylglycerol accumulation by its overexpression in the unicellular red alga Cyanidioschyzon merolae. Algal Res. 38, 101396. 10.1016/j.algal.2018.101396

[B52] Takeuchi T. Benning C. (2019). Nitrogen-dependent coordination of cell cycle, quiescence and TAG accumulation in *Chlamydomonas* . Biotechnol. Biofuels 12, 292. 10.1186/s13068-019-1635-0 31890020 PMC6927116

[B53] Tian Y. Lv X. Xie G. Zhang J. Xu Y. Chen F. (2018). Seed-specific overexpression of AtFAX1 increases seed oil content in Arabidopsis. Biochem. Biophys. Res. Commun. 500, 370–375. 10.1016/j.bbrc.2018.04.081 29654768

[B54] Tian Y. Lv X. Xie G. Wang L. Dai T. Qin X. (2019). FAX2 mediates fatty acid export from plastids in developing Arabidopsis seeds. Plant Cell Physiol. 60, 2231–2242. 10.1093/pcp/pcz117 31198959

[B55] Troncoso-Ponce M. A. Nikovics K. Marchive C. Lepiniec L. Baud S. (2015). New insights on the organization and regulation of the fatty acid biosynthetic network in the model higher plant *Arabidopsis thaliana* . Biochimie 120, 3–8. 10.1016/j.biochi.2015.05.013 26025475

[B56] Weber E. Engler C. Gruetzner R. Werner S. Marillonnet S. (2011). A modular cloning system for standardized assembly of multigene constructs. Plos One 6, e16765. 10.1371/journal.pone.0016765 21364738 PMC3041749

[B57] Xiao Z. Tang F. Zhang L. Li S. Wang S. Huo Q. (2021). The Brassica napus fatty acid exporter FAX1-1 contributes to biological yield, seed oil content, and oil quality. Biotechnol. Biofuels 14, 190. 10.1186/s13068-021-02035-4 34587987 PMC8482660

[B58] Xu C. Shanklin J. (2016). Triacylglycerol metabolism, function, and accumulation in plant vegetative tissues. Annu. Rev. Plant Biol. 67, 179–206. 10.1146/annurev-arplant-043015-111641 26845499

[B59] Young D. Y. Shachar-Hill Y. (2021). Large fluxes of fatty acids from membranes to triacylglycerol and back during N-deprivation and recovery in *Chlamydomonas* . Plant Physiol. 185, 796–814. 10.1093/plphys/kiaa071 33822218 PMC8133548

[B60] Zhu L. He S. Liu Y. Shi J. Xu J. (2020). Arabidopsis FAX1 mediated fatty acid export is required for the transcriptional regulation of anther development and pollen wall formation. Plant Mol. Biol. 104, 187–201. 10.1007/s11103-020-01036-5 32681357

