# Supplementary material for: Fatty acid export (FAX) proteins contribute to oil production in the green microalga Chlamydomonas reinhardtii
Source: Front Mol Biosci. 2022 Aug 30;9:939834. doi: 10.3389/fmolb.2022.939834 (PMC9470853; doi:10.3389/fmolb.2022.939834)
Supplement: Supplementary file 1 [file DataSheet2.pdf]

Table S1. List of oligonucleotides used in this study.

Base-pairing nucleotides of the amiRNA and *Cr-FAX1*, *Cr-FAX5* mRNAs (uppercase letters) are shaded in turquoise, mismatches in gray. The loop region of the amiRNA hairpin is underlined (compare **Figure 1A**).

name	sequence (5'-3')	application
CrFAX1gen-#1	TTGAAGACAAAATGGCTGCTTCCCTGC	domestication, L0 module *Cr-FAX1*
CrFAX1gen-#2	TTGAAGACGGTCGCAATGATAGCAGACGCGGGCA	
CrFAX1gen-#3	TTGAAGACTTGCGACCGACACTGATAATCAATGC	
CrFAX1gen-#4	TTGAAGACTCCGAACCCTCAGCCTTGCCGGCAGCCTTCTTCTTGG	
CrFAX5gen-#1	TTGAAGACAAAATGTATGACTTTTGCTTTTCGCCCATTTACTCGGTGTTCCTGGCCC	domestication, L0 module *Cr-FAX5*
CrFAX5gen-#2	TTGAAGACGTTTTCTCGAACCGCCTGTACATC	
CrFAX5gen-#3	TTGAAGACGAGAAAACGCACTCGGTACCCGCGGC	
CrFAX5gen-#4	TTGAAGACGAAGTCCGCAACACGTCCACGCCAACC	
CrFAX5gen-#5	TTGAAGACGAGACTTCGTATGTTGCGCCAGTCATACA	
CrFAX5gen-#6	TTGAAGACTCCGAACCGTGAGCCTTACCGTGCGCGGCGGGCTTGGGG	
CrFAX1-amiFor	ctagtGTGTTGCACGATTTCTCCATAtctcgctgatcggcaccatgggggtggtggtgatcagcgctaTATGCAGAAATCGTGCAACACg	amiRNA constructs *Cr-FAX1, Cr-FAX5*
CrFAX1-amiRev	ctagcGTGTTGCACGATTTCTGCATAtagcgctgatcaccaccacccccatggtgccgatcagcgagaTATGGAGAAATCGTGCAACACa	
CrFAX5-amiFor	ctagtAAGTACAGGCGGTTCGTGAAAtctcgctgatcggcaccatggggtggtggtgatcagcgctaTTTCTCGAACCGCCTGTACTTg	
CrFAX5-amiRev	ctagcAAGTACAGGCGGTTCGAGAAAtagcgctgatcaccaccacccccatggtgccgatcagcgagaTTTCACGAACCGCCTGTACTTa	

FIGURE S1 | Growth of UVM4 wild-type Chlamydomonas cells.

Growth of Chlamydomonas UVM4 cells was started in TAP medium by inoculating four independent 100 ml cultures to an OD_{750} of 0.05 with a freshly grown preculture. Growth was followed by determination of the OD_{750} **(A)** and the cell number **(B)** at the indicated time points (n=4 ± SD). Cell number was measured by a TC20 automated cell counter (Bio-Rad). Grey rectangles indicate mid-log growth phase for harvesting of cells.

FIGURE S2 | Comparison of FAX proteins in Chlamydomonas and Arabidopsis.

(A) Arabidopsis and Chlamydomonas FAX3 and FAX7 proteins. Sequence alignment of mature FAX3 and FAX7 proteins in Chlamydomonas and Arabidopsis. The four membrane-embedded α-helices (red boxes) within the Tmemb_14 domain (pfam PF03647 motif, green line) are depicted according to the Aramemnon consensus prediction AramTmCon for At-FAX3 (Schwacke et al., 2003). Processing sites for stromal peptidases according to TargetP2.0 (Almagro Armenteros et al., 2019) are indicated by green triangles. The FAX3 specific N-terminal poly glycine motif (poly G) is specified. Among the FAX-protein family in Arabidopsis, Cr-FAX3 shows highest similarity to At-FAX3 (24% aa identity) and Cr-FAX7 is most likely related to At-FAX7 (28% identical aas). Names, gene codes (AGI, Phytozome), and protein IDs (UniProt) are as follows: Cr-FAX3 (Cre08.g383300, A0A2K3DI83), At-FAX3 (At3g43520, Q94A32 [please note that in UniProt this sequence is annotated as FAX2]), Cr-FAX7 (Cre09.g387838, A0A2K3DDV7), At-FAX7 (At2g26240, O64847).

(B) Phylogenetic guide tree of all FAX proteins found in Arabidopsis and Chlamydomonas. For the plastid-predicted FAX1-FAX4 proteins mature protein sequences were used. The tree was created by ClustalW.

FIGURE S3 | Cr-FAX1 and Cr-FAX5 localize to intracellular membranes of different origin. **(A)** Immunoblot analysis of Cr-FAX1 and Cr-FAX5 in membrane protein extracts from wild-type Chlamydomonas cells. Intact UVM4 *C. reinhardtii* cells (whole cell, wc) were broken up by freeze/thaw cycles and fractionated into membrane pellet (mp) and supernatant (sup) by centrifugation. 10 µg of proteins were separated by SDS-PAGE and subjected to immunoblot analysis using antisera directed against Cr-FAX1 and Cr-FAX5. As control we used α-Lhcb4 for detection of thylakoid CP29 chlorophyll a/b binding protein of PSII from higher plants.
(B) Immunoblot analysis of Cr-FAX1 and Cr-FAX5 in fractionated chloroplasts and microsomes from wild-type Chlamydomonas cells. Intact UVM4 *C. reinhardtii* cells from **(A)** were fractionated into chloroplasts (c), microsomes (ms) and supernatant (sup) by differential centrifugation. 20 µg of proteins were separated by SDS-PAGE and subjected to immunoblot analysis using antisera directed against Cr-FAX1 and Cr-FAX5. Antisera directed against the ER-membrane intrinsic ABC transporter ABCA2 (Jang et al., 2020), BiP (binding immunoglobulin protein, ER lumen), and Lhcb4 (thylakoid membranes) served as controls. The faint signal of α-Cr-FAX5 at around 14 kDa (arrow head) most likely represents a cross reaction with Cr-FAX1, whereas the weak signals of α-Cr-ABCA2 and α-BiP in chloroplasts don't have the correct molecular mass and thus probably correspond to unknown background. Please note that the signal of the BiP antiserum in the microsomal fraction (asterisk) has the apparent correct size of 80 kDa as annotated by the Agrisera datasheet AS09 481. For the BiP antiserum signals of other lanes on the same blot, which are not subject of this study, have been removed as indicated by gray, dotted lines. Numbers indicate molecular mass of proteins in kDa.

FIGURE S4 | Controls for immunofluorescence assays.

(A) Unspecific *in situ* immunofluorescence signals (TRITC background fluorescence) of α-HA in Chlamydomonas cells. Intact, fixed wild-type UVM4 *C. reinhardtii* cells were treated with antiserum against an HA-tag and unspecific signals of the secondary antibody were detected by coupled fluorescence of TRITC (tetramethylrhodamine-isothiocyanate). Shown are pseudo transmission images (PTI), TRITC-fluorescence (green), chlorophyll fluorescence (Chl) and an overlay of both. A cartoon (top) illustrates positioning and organelles of the respective Chlamydomonas cells. TRITC fluorescence was exited at 561 nm and recorded between 565-583 nm, chlorophyll: excitation at 458 nm, emission at 626-735 nm by a Leica LSM780 confocal microscope. Please note that chlorophyll signals were enhanced to detect residual fluorescence after fixation and washing steps for antiserum treatment. c; chloroplast, cw; cell wall, n; nucleus, p; pyrenoid.

(B) Schematic drawing of level 1 constructs according to the MoClo syntax (Patron et al., 2015; Crozet et al., 2018) for chloroplast (C) and ER-targeting of mVenus fluorescence controls. The following level 0 modules were assembled in destination vector pICH47742 (Weber et al., 2011).

mVenus (ER): *HSP70A-RBCS2*, hybrid promotor of HSP70A heat shock protein 70A and rubisco small subunit 2 (position A1-B2); SP BIP2, signal peptide of ER lumen *"binding immunoglobulin protein 2"* (B2); **mVenus**, mVenus (i2) with second intron of *RBCS* (B3-B4); 3xHA-KDEL, HA-tag with "KDEL" ER retention signal (B5); *RPL23*ter, terminator of 50S ribosomal protein L23 (B6-C1).

mVenus (C): *HSP70A-RBCS2*, hybrid promotor of HSP70A heat shock protein 70A and rubisco small subunit 2 (position A1-B2); cTP USPA, chloroplast targeting peptide of universal stress protein A (B2); **mVenus**, mVenus (i2) with second intron of *RBCS* and stop codon (B3-B5); *RPL23*ter, terminator of 50S ribosomal protein L23 (B6-C1).

FIGURE S5 | Inducible knockdown of *Cr-FAX1* and *Cr-FAX5* in cw15-325 Chlamydomonas cells.

Immunoblot analysis of Cr-FAX1 **(A)** and Cr-FAX5 **(B)** in protein extracts from Chlamydomonas cells that were transformed with amiRNA knockdown constructs for Cr-FAX1 (kd-C) and for Cr-FAX5 (kd-E) under control of the inducible *NIT1* promoter (compare **Figure 1A**). Samples were collected prior to *NIT1* induction (d0) and one (d1) as well as two days (d2) after a change of the nitrogen source in TAP medium from ammonium to nitrate for induction of *NIT1*. Proteins were extracted from cells of independently derived *Cr-FAX1* and *Cr-FAX5* knockdown strains as well as the respective untransformed parental strain cw15-325 (pl). Equal amounts of proteins (isolated from the same quantity of cells corresponding to 2 µg chlorophyll, exception lowest panel in **(B)**, see below) were separated by SDS-PAGE and subjected to immunoblot analysis using antisera directed against Cr-FAX1**(A)** and Cr-FAX5 **(B)**. Numbers indicate molecular mass of proteins in kDa. Note that kd-C#22, kd-C#24, and kd-E#22, kd-E#28 we observed a strong reduction of Cr-FAX1 and Cr-FAX5 proteins already prior to *NIT1* induction at d0 (arrows), most likely , the strains co-C#12 in **(A)**, co-E#4 in **(B)** showed no knockdown effect at d0 (asterisks) and thus were used as control (co) lines. Note that for the "co-E#4 blot" (**[B]**, lowest panel), non-equal amounts of protein were loaded for kd-C#12, kd-C#3. Therefore, on this blot, co-E#4 can only be compared to the pl sample content as indicated by arrows.

FIGURE S6 | Polar lipids, total lipids and distribution of FAs in total lipids of knockdown lines.

(A) Knockdown of ER-localized Cr-FAX5 decreases ER-produced DGTS in Chlamydomonas cells. Two independently generated *C. reinhardtii* strains each for knockdown of Cr-FAX1: lines kd-C#22 (green) and kd-C#24 (light green), and Cr-FAX5: lines kd-E#22 (blue) and kd-E#28 (light blue) as well as the respective control lines with wild-type levels for Cr-FAX1 (co-C#12; white) and Cr-FAX5 (co-E#4; gray) were grown in TAP medium and harvested in the exponential growth phase for determination of polar lipids via thin layer chromatography (TLC). Polar lipids [µg/10^6 cells] were quantified densitometrically after separation by TLC from five individual liquid cultures of each strain ($n = 5 \pm SD$). P-values for significantly different lipid content when compared to the respective control strains (co-C#12 for Cr-FAX1kd, co-E#4 for Cr-FAX5kd) are indicated (double-sided student t-test): * $p<0.05$.
DGDG, digalactosyl-diacylglycerol; DGTS, diacylglyceryl-trimethyl-homoserine; MGDG, monogalactosyl-diacylglycerol; PE, phosphatidylethanolamine; PG, phosphatidylglycerol; PI, phosphatidylinositol; SQGD, sulfoquinovosyl-diacylglycerol; TAG, triacyl-glycerol.

(B) Total lipids [µg/10^6 cells, $n = 5 \pm SD$] in *Cr-FAX1*, *Cr-FAX5* knockdown and control strains as described in **(A)**.

(C) FA composition in total lipids from *Cr-FAX1*, *Cr-FAX5* knockdown and control strains described in **(A)** is given in relation to total lipid content [%; $n = 5 \pm SD$]. Numbers in brackets indicate positions of desaturated C=C bonds.

FIGURE S7 | Overexpression of *Cr-FAX1* and *Cr-FAX5* in UVM4 Chlamydomonas cells.

(A) Immunoblot analysis of Cr-FAX1 (left) and Cr-FAX5 (right) in protein extracts from Chlamydomonas cells that were transformed with overexpression constructs for *Cr-FAX1* (ox-C) and *Cr-FAX5* (ox-E) under control of the constitutive *PSAD* promoter (compare **Figure 1B**). Proteins were extracted from cells of independently derived Cr-FAX1ox (left) and Cr-FAX5ox strains (right), as well as the respective untransformed, parental strain UVM4. Equal amounts of proteins (isolated from the same quantity of cells corresponding to 2 µg chlorophyll) were separated by SDS-PAGE and subjected to immunoblot analysis using antisera directed against Cr-FAX1 or Cr-FAX5. Numbers indicate molecular mass of proteins in kDa.

Note that the strains ox-C#8, C#12 and ox-E#1, E#8 show overexpression of Cr-FAX1, Cr-FAX5, respectively, as indicated by arrows. Therefore, we used these strains in all following experiments.

(B) Overexpression of *Cr-FAX1* does not affect Cr-FAX5, and *Cr-FAX5*ox does not change Cr-FAX1 protein levels. Immunoblot analysis of Cr-FAX5 (left) and Cr-FAX1 (right) in protein extracts from Cr-FAX1ox lines (left) and Cr-FAX5ox lines (right) as described in **(A)**.

FIGURE S8 | Overexpression of Cr-FAX5 affects polar lipid content and FA distribution in Chlamydomonas.**(A)** Overexpression of ER-localized Cr-FAX5 decreases plastid-derived glycolipids in Chlamydomonas cells. Two independently generated *C. reinhardtii* strains each for overexpression of Cr-FAX1: lines ox-C#8 (green) and ox-C#12 (light green), and Cr-FAX5: lines ox-E#1 (blue) and ox-E#8 (light blue) as well as the respective wild-type parental strain UVM4 (white) were grown in TAP medium and harvested in the exponential growth phase for determination of polar lipids via TLC. Polar lipids [µg/10^6 cells] were quantified densitometrically after separation by TLC from five individual liquid cultures of each strain (n = 5 ± SD). P-values for significantly different lipid content when compared to UVM4 are indicated (double-sided student t-test): * $p<0.05$.
DGDG, digalactosyl-diacylglycerol; DGTS, diacylglyceryl-trimethyl-homoserine; MGDG, monogalactosyl-diacylglycerol; PE, phosphatidylethanolamine; PG, phosphatidylglycerol; PI, phosphatidylinositol; SQGD, sulfoquinovosyl-diacylglycerol; TAG, triacyl-glycerol.
(B) Total lipids [µg/10^6 cells, n = 5 ± SD] in *Cr-FAX1*, *Cr-FAX5* overexpression and UVM4 parental strains as described in **(A)**.
(C) FA composition in total lipids from *Cr-FAX1*, *Cr-FAX5* overexpression and UVM4 parental strains described in **(A)** is given in relation to total lipid content [%; n = 5 ± SD]. Numbers in brackets indicate positions of desaturated C=C bonds. P-values for significantly different FA ratios when compared to UVM4 are indicated (double-sided student t-test): *** $p<0.001$; ** $p<0.01$.

FIGURE S9 | Knockdown and overexpression of Cr-FAX1, Cr-FAX5 does not affect growth of cultures at standard conditions.

(A) Growth of the respective knockdown lines for *Cr-FAX1* and *Cr-FAX5* as well as of corresponding control strains was followed for 4 days in TAP medium. Cell density was determined by OD at 750 nm.

Growth of the respective overexpression lines for *Cr-FAX1* and *Cr-FAX5* as well as of corresponding wild type UVM4 was followed for 4 days in TAP medium. Cell density was determined by OD at 750 nm.

References

Almagro Armenteros, J.J., Salvatore, M., Emanuelsson, O., Winther, O., von Heijne, G., Elofsson, A., et al. (2019). Detecting sequence signals in targeting peptides using deep learning. *Life Science Alliance* 2, e201900429.

Crozet, P., Navarro, F.J., Willmund, F., Mehrshahi, P., Bakowski, K., Lauersen, K.J., et al. (2018). Birth of a Photosynthetic Chassis: A MoClo Toolkit Enabling Synthetic Biology in the Microalga Chlamydomonas reinhardtii. *ACS Synth Biol* 7, 2074-2086.

Jang, S., Kong, F., Lee, J., Choi, B.Y., Wang, P., Gao, P., et al. (2020). CrABCA2 Facilitates Triacylglycerol Accumulation in Chlamydomonas reinhardtii under Nitrogen Starvation. *Mol Cells* 43, 48-57.

Patron, N.J., Orzaez, D., Marillonnet, S., Warzecha, H., Matthewman, C., Youles, M., et al. (2015). Standards for plant synthetic biology: a common syntax for exchange of DNA parts. *New Phytol* 208, 13-19.

Schwacke, R., Schneider, A., van der Graaff, E., Fischer, K., Catoni, E., Desimone, M., et al. (2003). ARAMEMNON, a novel database for Arabidopsis integral membrane proteins. *Plant Physiol* 131, 16-26.

Weber, E., Engler, C., Gruetzner, R., Werner, S., and Marillonnet, S. (2011). A modular cloning system for standardized assembly of multigene constructs. *Plos One* 6, e16765.
