# Supplementary material for: Fatty acid export (FAX) proteins contribute to oil production in the green microalga Chlamydomonas reinhardtii
Source: Front Mol Biosci. 2022 Aug 30;9:939834. doi: 10.3389/fmolb.2022.939834 (PMC9470853; doi:10.3389/fmolb.2022.939834)
Supplement: Supplementary file 2 [file DataSheet1.pdf]

Figure S1

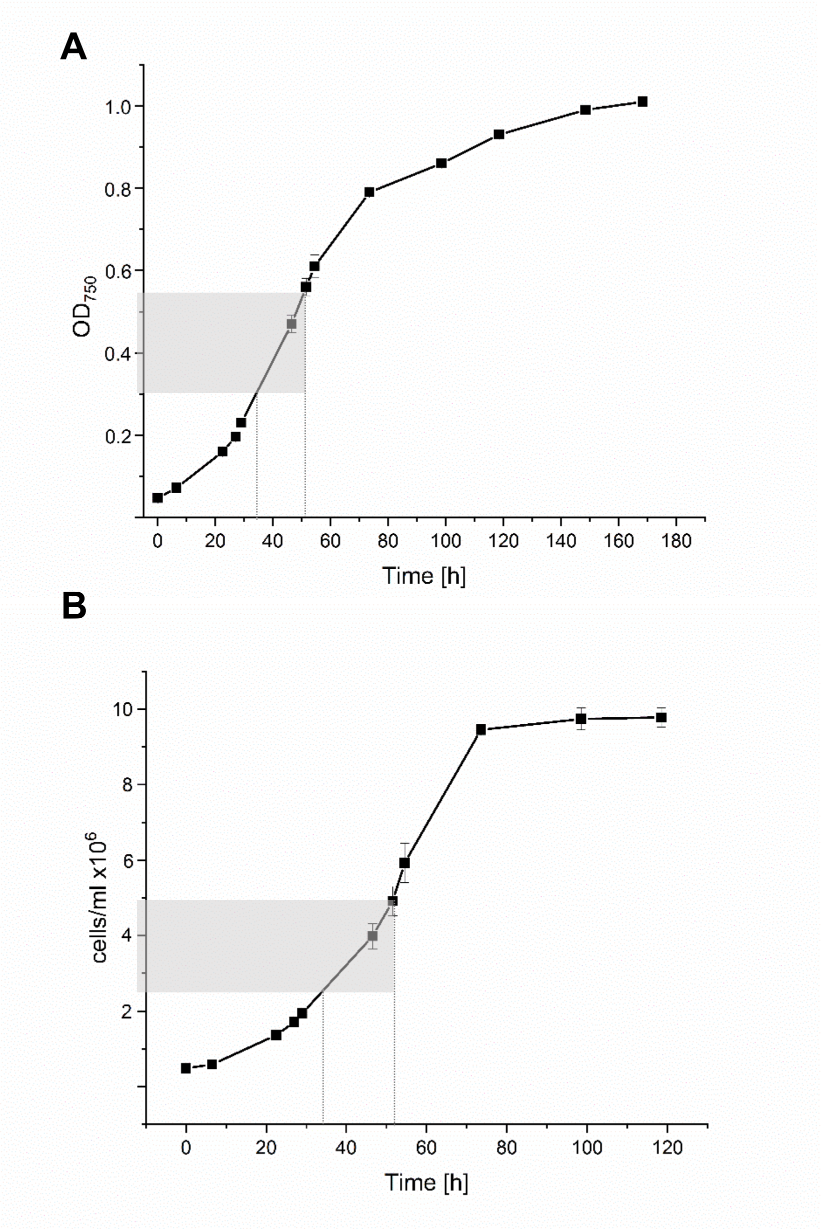
A
1.0
0.8
0.6
0.4
0.2
OD_{750}
0
20
40
60
80
100
120
140
160
180
Time [h]
B
10
8
6
4
2
cells/ml x10^6
0
20
40
60
80
100
120
Time [h]

Figure S2

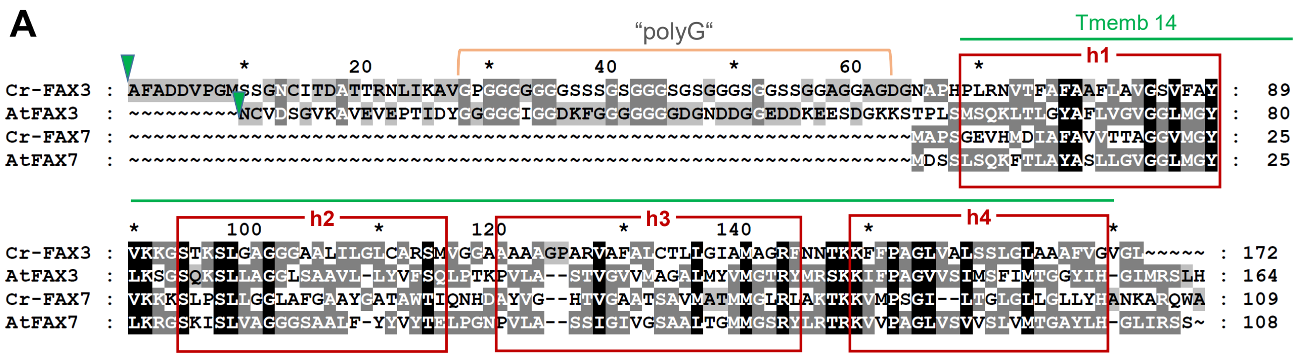
A
"polyG"
Tmemb 14
h1
h2
h3
h4
Cr-FAX3
AtFAX3
Cr-FAX7
AtFAX7

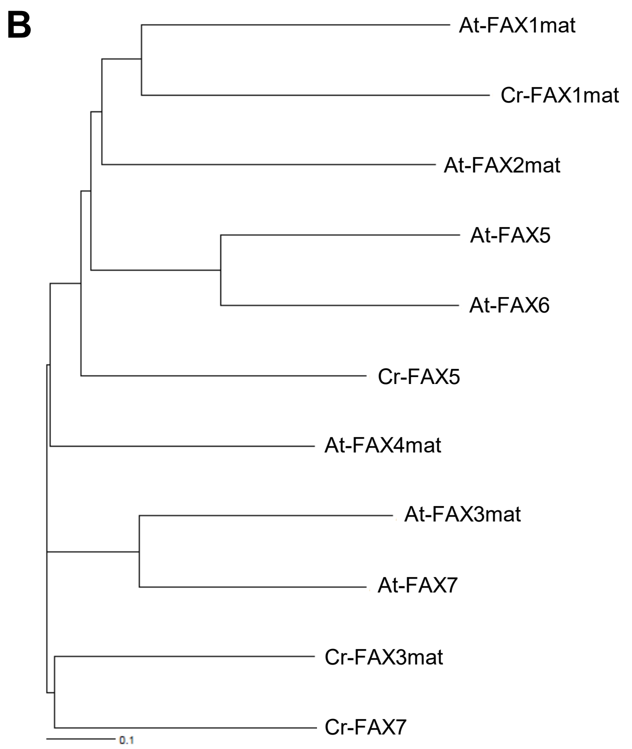
B
At-FAX1mat
Cr-FAX1mat
At-FAX2mat
At-FAX5
At-FAX6
Cr-FAX5
At-FAX4mat
At-FAX3mat
At-FAX7
Cr-FAX3mat
Cr-FAX7
0.1

Figure S3

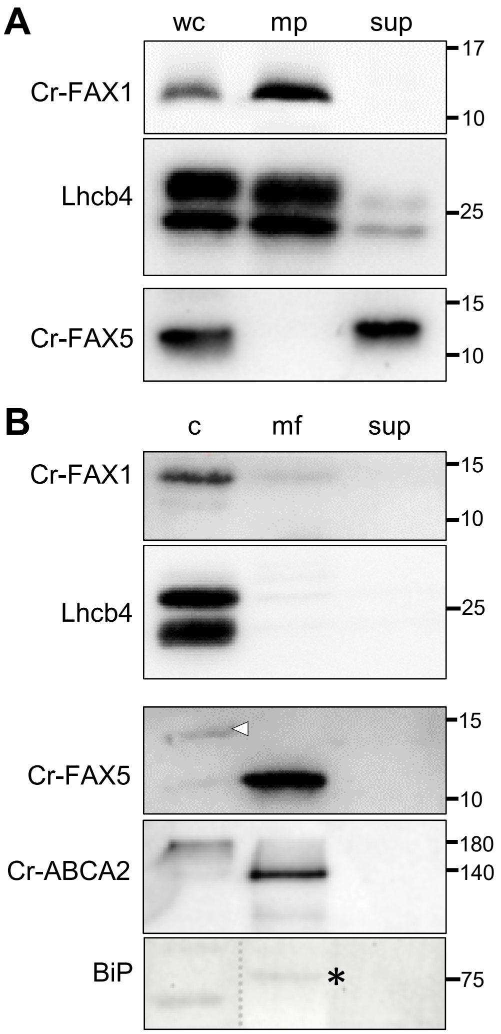

A
wc
mp
sup
Cr-FAX1
Lhcb4
Cr-FAX5
17
10
25
15
10
B
c
mf
sup
Cr-FAX1
Lhcb4
Cr-FAX5
Cr-ABCA2
BiP
15
10
25
15
10
180
140
75
*

Figure S4

A

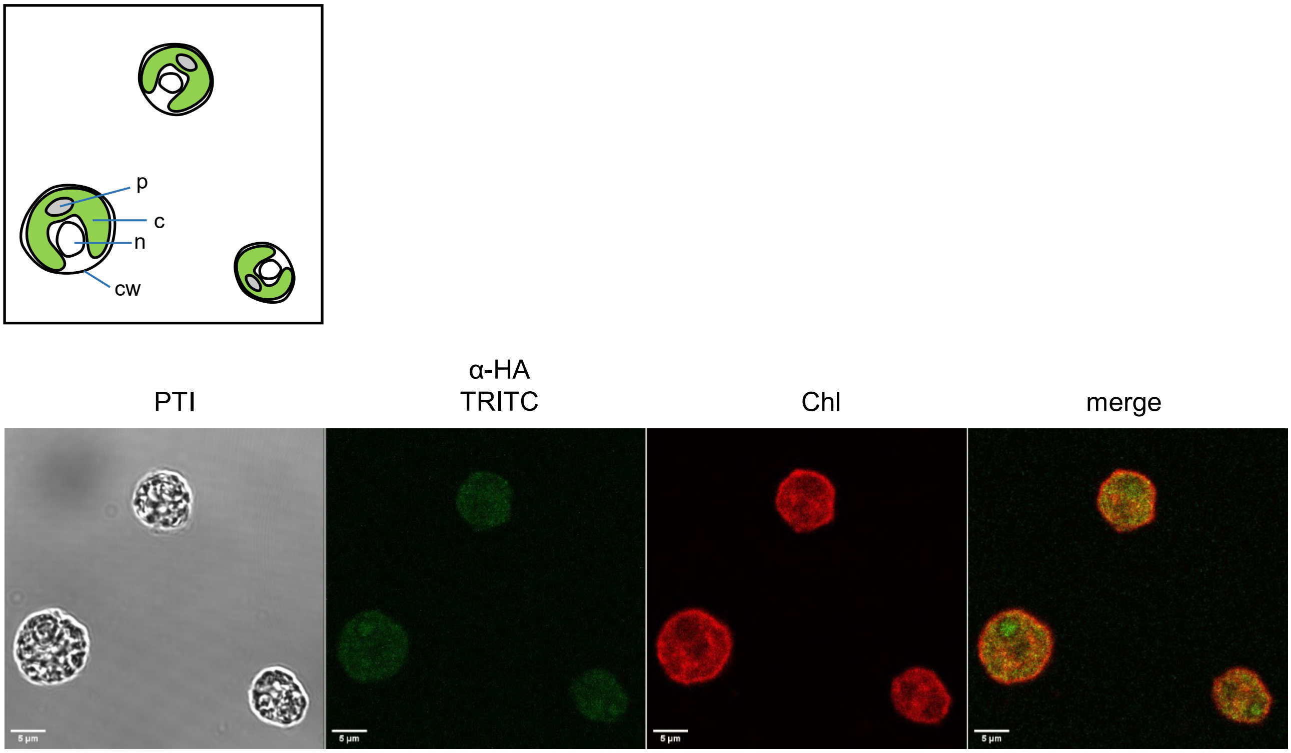

B

		promoter						5'UTR		5'UTR/CDS		CDS						3'UTR + Ter			
	**1**	**A1**	**2**	**A2**	**3**	**A3**	**4**	**B1**	**5**	**B2**	**6**	**B3**	**7**	**B4**	**8**	**B5**	**9**	**B6**	**10**	**C1**	**11**
**mVenus (ER)**	**1**	*HSP70A-RBCS2*							**5**	SP BIP2	**6**	**mVenus**			**8**	3XHA-KDEL	**9**	*RPL23*ter			**11**
**mVenus (C)**	**1**	*HSP70A-RBCS2*							**5**	cTP USPA	**6**	**mVenus** + STOP					**9**	*RPL23*ter			**11**

Figure S5

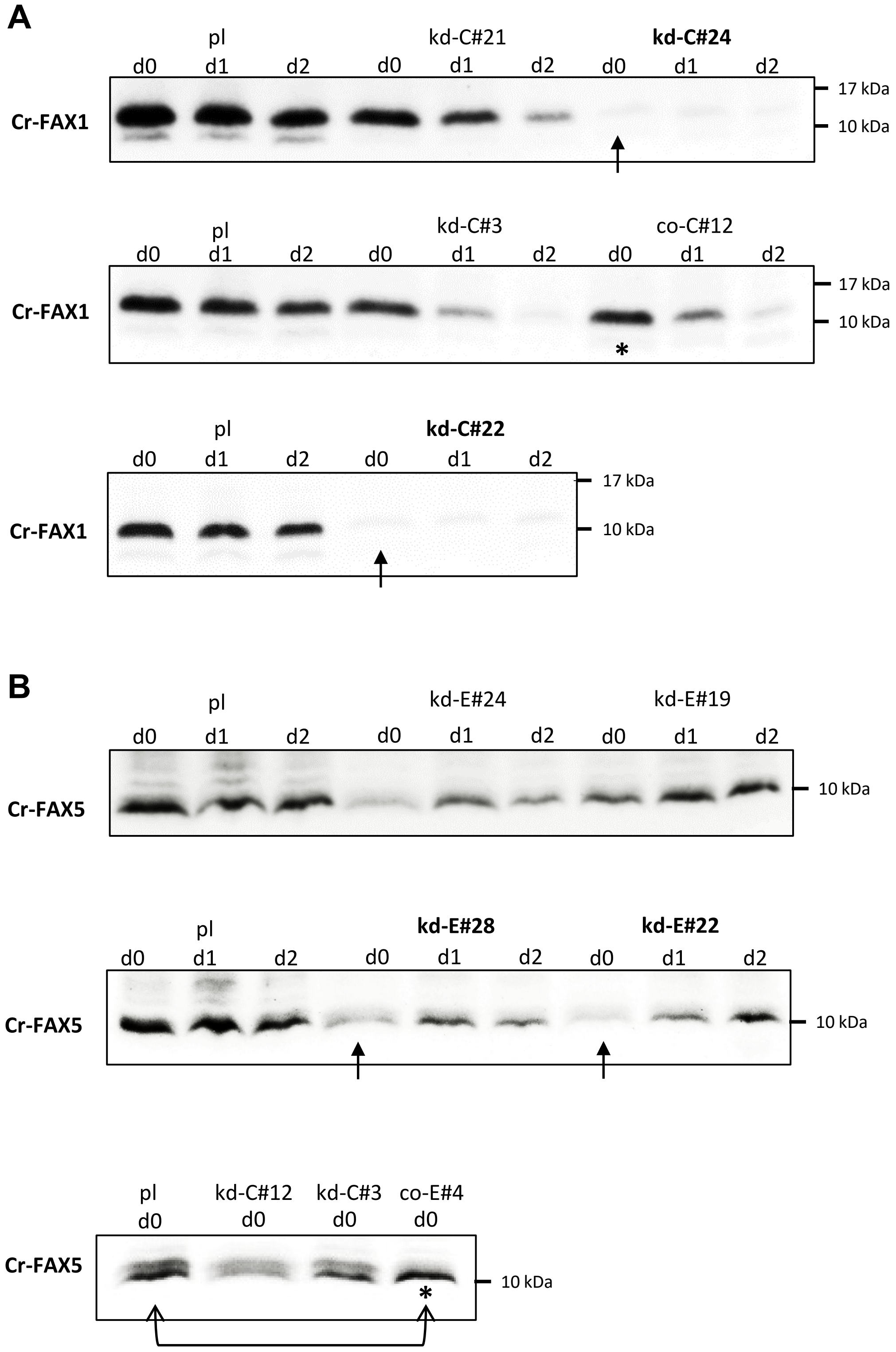
A
pl
kd-C#21
kd-C#24
d0 d1 d2 d0 d1 d2 d0 d1 d2
Cr-FAX1
17 kDa
10 kDa
pl
kd-C#3
co-C#12
d0 d1 d2 d0 d1 d2 d0 d1 d2
Cr-FAX1
17 kDa
10 kDa
*
pl
kd-C#22
d0 d1 d2 d0 d1 d2
Cr-FAX1
17 kDa
10 kDa
B
pl
kd-E#24
kd-E#19
d0 d1 d2 d0 d1 d2 d0 d1 d2
Cr-FAX5
10 kDa
pl
kd-E#28
kd-E#22
d0 d1 d2 d0 d1 d2 d0 d1 d2
Cr-FAX5
10 kDa
pl
kd-C#12
kd-C#3
co-E#4
d0 d0 d0 d0
Cr-FAX5
10 kDa
*

Figure S6

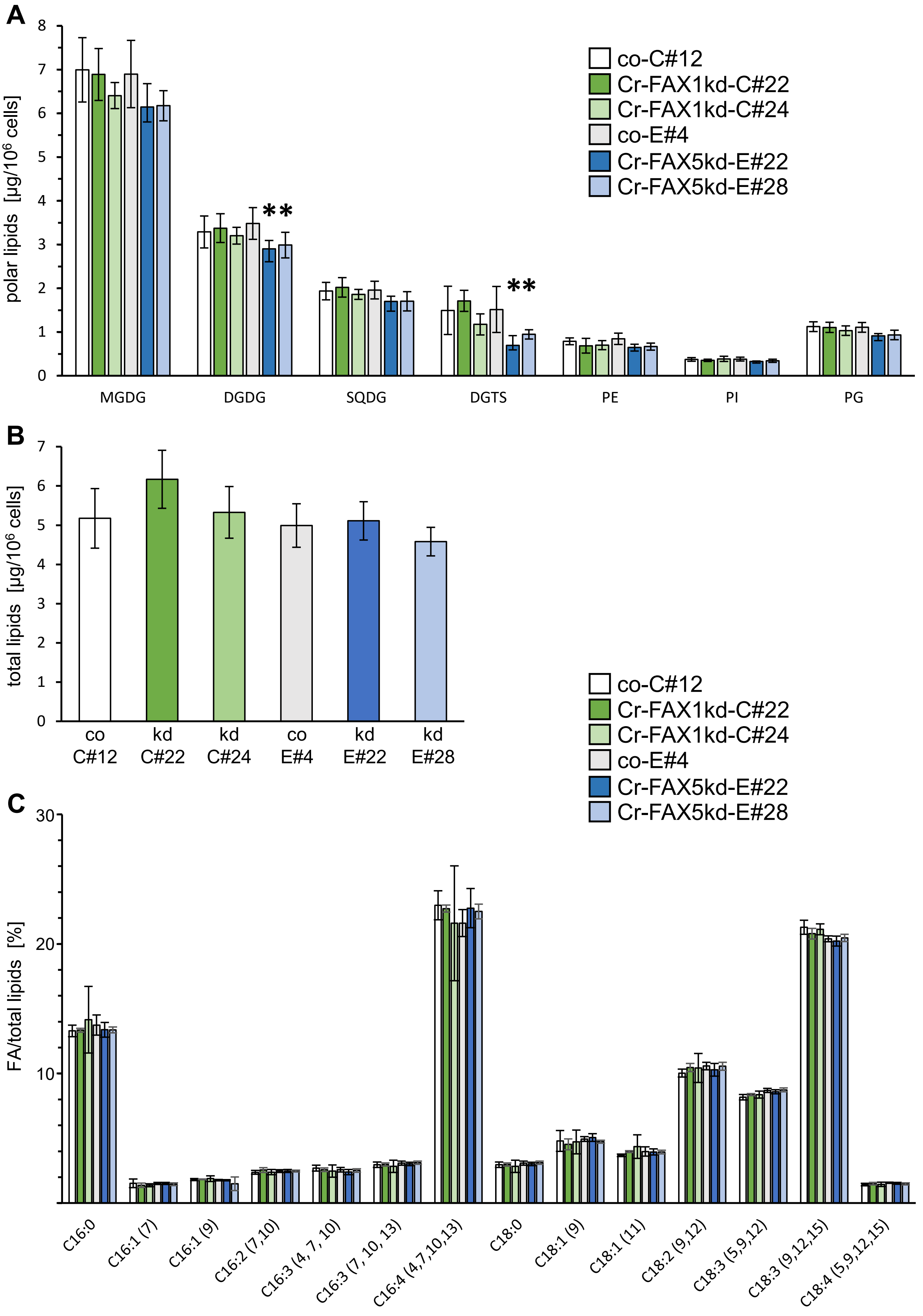
A
polar lipids [µg/10^6 cells]
8
7
6
5
4
3
2
1
0
**
**
MGDG
DGDG
SQDG
DGTS
PE
PI
PG
co-C#12
Cr-FAX1kd-C#22
Cr-FAX1kd-C#24
co-E#4
Cr-FAX5kd-E#22
Cr-FAX5kd-E#28
B
total lipids [µg/10^6 cells]
7
6
5
4
3
2
1
0
co C#12
kd C#22
kd C#24
co E#4
kd E#22
kd E#28
co-C#12
Cr-FAX1kd-C#22
Cr-FAX1kd-C#24
co-E#4
Cr-FAX5kd-E#22
Cr-FAX5kd-E#28
C
FA/total lipids [%]
30
20
10
C16:0
C16:1 (7)
C16:1 (9)
C16:2 (7,10)
C16:3 (4, 7, 10)
C16:3 (7, 10, 13)
C16:4 (4,7,10,13)
C18:0
C18:1 (9)
C18:1 (11)
C18:2 (9,12)
C18:3 (5,9,12)
C18:3 (9,12,15)
C18:4 (5,9,12,15)

Figure S7

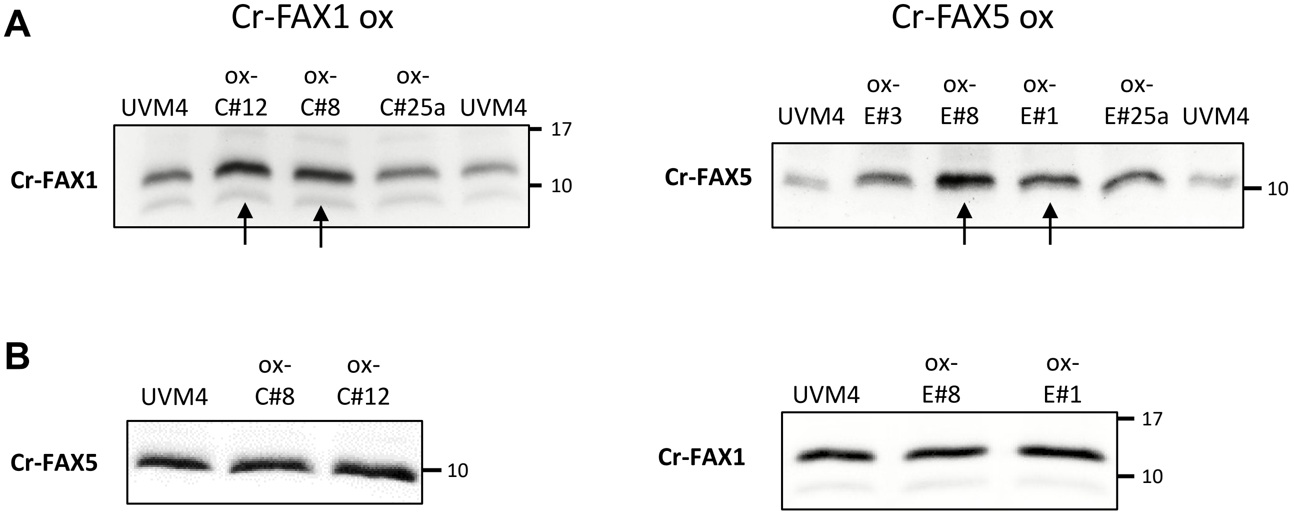
A
Cr-FAX1 ox
UVM4
ox-C#12
ox-C#8
ox-C#25a
UVM4
Cr-FAX1
17
10
Cr-FAX5 ox
UVM4
ox-E#3
ox-E#8
ox-E#1
ox-E#25a
UVM4
Cr-FAX5
10
B
UVM4
ox-C#8
ox-C#12
Cr-FAX5
10
UVM4
ox-E#8
ox-E#1
Cr-FAX1
17
10

Figure S8

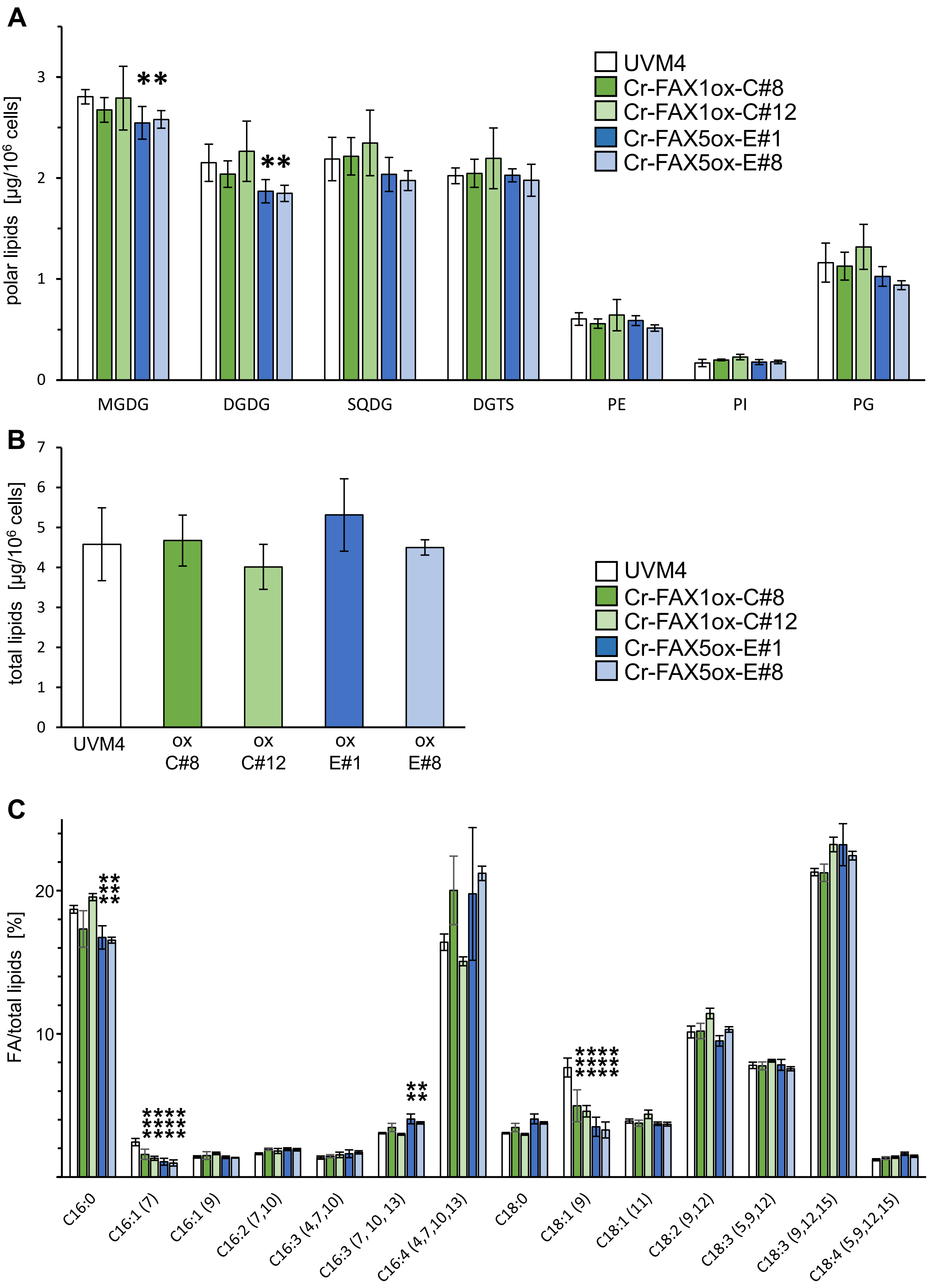

A
polar lipids [µg/10⁶ cells]
MGDG
DGDG
SQDG
DGTS
PE
PI
PG
UVM4
Cr-FAX1ox-C#8
Cr-FAX1ox-C#12
Cr-FAX5ox-E#1
Cr-FAX5ox-E#8
B
total lipids [µg/10⁶ cells]
UVM4
ox C#8
ox C#12
ox E#1
ox E#8
UVM4
Cr-FAX1ox-C#8
Cr-FAX1ox-C#12
Cr-FAX5ox-E#1
Cr-FAX5ox-E#8
C
FA/total lipids [%]
C16:0
C16:1 (7)
C16:1 (9)
C16:2 (7,10)
C16:3 (4,7,10)
C16:3 (7, 10, 13)
C16:4 (4,7,10,13)
C18:0
C18:1 (9)
C18:1 (11)
C18:2 (9,12)
C18:3 (5,9,12)
C18:3 (9,12,15)
C18:4 (5,9,12,15)

Figure S9

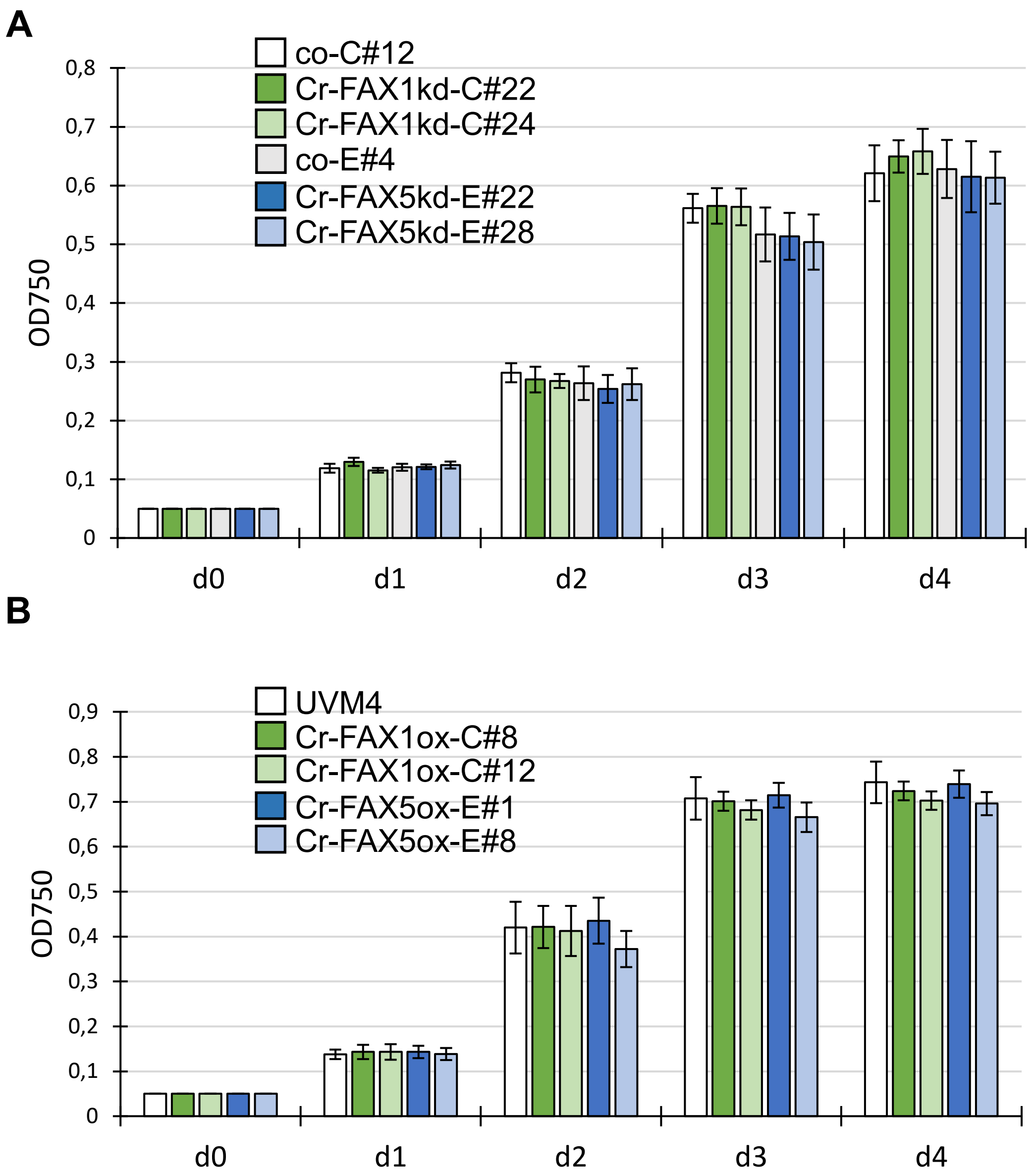
A
co-C#12
Cr-FAX1kd-C#22
Cr-FAX1kd-C#24
co-E#4
Cr-FAX5kd-E#22
Cr-FAX5kd-E#28
OD750
0,8
0,7
0,6
0,5
0,4
0,3
0,2
0,1
0
d0
d1
d2
d3
d4
B
UVM4
Cr-FAX1ox-C#8
Cr-FAX1ox-C#12
Cr-FAX5ox-E#1
Cr-FAX5ox-E#8
OD750
0,9
0,8
0,7
0,6
0,5
0,4
0,3
0,2
0,1
0
d0
d1
d2
d3
d4
